# Nanolasers: More than a decade of progress, developments and challenges

**DOI:** 10.1515/nanoph-2023-0369

**Published:** 2024-04-15

**Authors:** Thomas Charles Ellis, Sahand Eslami, Stefano Palomba

**Affiliations:** School of Physics, The University of Sydney, Sydney, Australia

**Keywords:** nanolaser, nanolaser category, nanolaser array, nanolaser applications, plasmonics, thresholdless lasing

## Abstract

As the demand for smaller and more compact lasers increases, the physical dimensions of laser diodes are already at the diffraction limit, which impairs this miniaturization trend and limits direct laser integration into photonic and especially nanophotonic circuits. However, plasmonics has allowed the development of a novel class of lasers that can be manufactured without being limited by diffraction, exhibiting ultralow energy consumption, small volumes, and high modulation speeds that could someday compete with their modern macroscale counterparts. Nevertheless, a wide variety of issues create roadblocks for further development and commercial adoption. Here we conduct a monolithic review in which we formulate the definition of a nanolaser, categorize nanolasers, and examine their properties and applications to determine if nanolasers do present a potential technological revolution as they seem to exhibit or are too restricted by the issues that plague them to ever succeed.

## 1 Introduction

The size imposed by the diffraction limit has constrained the dimensions of laser cavities to hundreds of nanometers, severely impairing the miniaturization trend seen in the previous decades. This all changed when in 2009 a few works were released in the literature presenting novel plasmonic nanolaser platforms that could confine light to sub diffraction limit scales. These works sparked a boom in novel nanolaser architectures and experimental demonstration not only exhibiting dimensions well beyond the diffraction limit but also showing intriguing operational characteristics.

With the plethora of new devices now under the banner of “nanolasers” it becomes a laborious task to find, group and study nanolaser devices with no current clear-cut definition of what a nanolaser physically represents. For example, a subwavelength photonic nanocuboid structure with a side length of 400 nm and volume of 0.49*λ* ^3^ was recently demonstrated lasing in air [1]. Although the nanocuboid laser does exhibit lasing with dimensions that lay in the subwavelength regime, it does not exhibit lasing beyond the diffraction limit, and, strictly speaking, its dimensions are still above the nano scale; therefore, can it be called a nanolaser? Another example can be represented by photonic crystal lasers. Such lasers generally consist of a 2D slab lattice, although 1D configurations are possible, thanks to an embedded point defect. They operate via total internal reflection and photonic bandgap light trapping. Although their individual structures approach the nanometer scale, the laser mode is much larger and their operation requires the whole lattice to work together – can this be still called a nanolaser?

To answer these questions, this review attempts to categorize and review recent advancement in nanolasers, starting by defining what nanolaser is. We will proceed by outlining their configurations, respective properties, demonstrated performance and potential applications, finally supplying our perspective if nanolasers are a technological revolution or a dead end.

## 2 What is a nanolaser?

Depending on the context, we have found in the literature that a nanolaser may refer to a device on the scale of tens of nanometers, hundreds of nanometers, or even thousands of nanometers with no strict guide on what is defined as a nanolaser. This can make the study of the field cumbersome and in some circumstances confusing. Here, a definition of nanolasers is proposed with the aim of easing future investigations by providing an appropriate framework.

“The nanoscale, based on the nanometer (nm) or one-billionth of a meter, exists specifically between 1 and 100 nm” [2]. In a more general sense, “materials with at least one dimension below one micron but greater than 1 nm can be considered nanoscale materials” [2]. Although we agree with these definitions, we consider the latter to be too relaxed and the former perhaps too strict.

**Figure 1:**
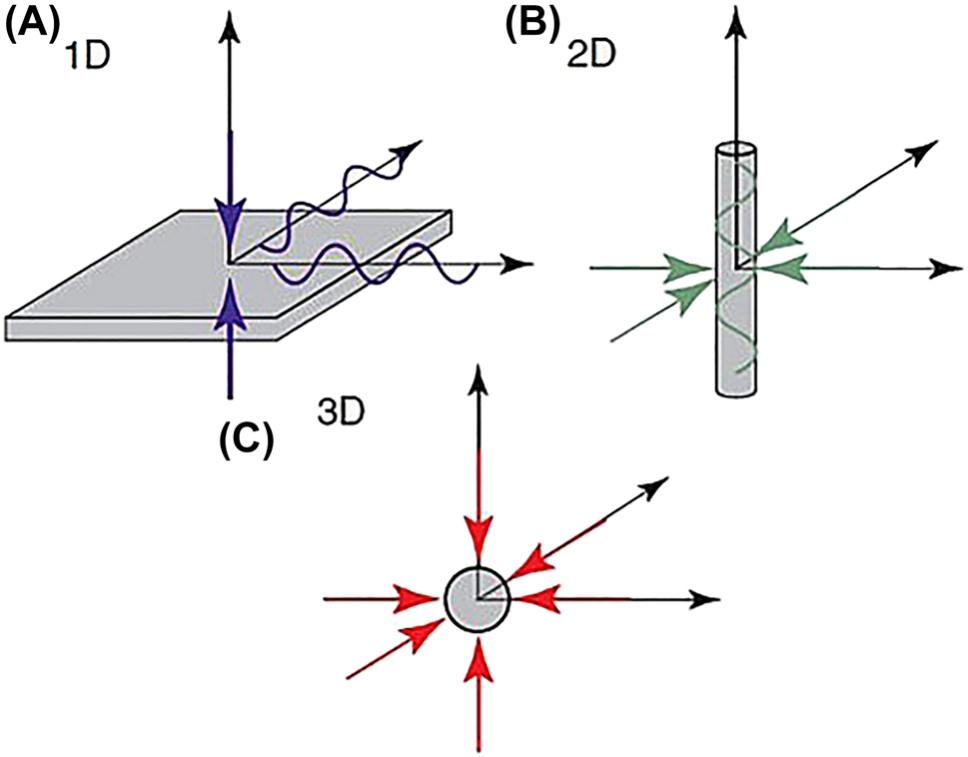
Various nanolaser confinements. (A) Prototype 1D nanolaser; (B) prototype 2D nanolaser; (C) prototype 3D nanolaser; showing confinement dimensions [3]. Reproduced with permission. [© 2019 WILEY-VCH Verlag GmbH & Co. KGaA, Weinheim].

Imposing a size limit on what can be defined as a nanolaser is controversial. One could use the strict definition of nanolaser, traditionally 100 nm or less, however this excludes all known photonic devices and the majority of plasmonic devices. Another method could be to base the limit on whether the device in question breaks the diffraction limit. This leads to a larger scale in the definition and would also permit devices that lase in the terahertz spectrum region featuring dimensions of many microns in scale being classified as a nanolaser. A more appropriate scale could be based on considering the effective length *L*_eff_ in a particular direction, which therefore includes the evanescent field, divided by two times the effective refractive index *n*, i.e. *L*_eff_/2*n*_eff_. However, even this definition leads to a scale that would also include larger dimensions for photonics devices and not well suited for comparing photonic-based with plasmonic-based nanolasers. Consequently, here we recognized the strict nanoscale definition (1–100 nm) is still appropriate to identify nanoscale objects, but we have arbitrarily extended to <400 nm in order to include most of the known photonic and plasmonic devices; we feel that above 400 nm, an object would be more appropriately defined as a sub-micron rather than a nanoscale object. In conclusion, we define “nanoscale confinement” the confinement of light in at least one spatial dimension, within the upper limit previously defined, i.e. <400 nm. Consequently, within this review we define *a nanolaser to be a device that is physically limited to the nanoscale (<400 nm) in at least one spatial dimension and generates laser light*. Furthermore, we classify nanolasers to belong to one of the following subcategories: 1D nanolaser, where light is confined to the nanoscale by one spatial dimension; 2D nanolaser, where light is confined to the nanoscale by two spatial dimensions; 3D nanolaser, where light is confined to the nanoscale by three spatial dimensions. As examples, a 1D nanolaser could be a plane where light is confined within the smallest dimension at the nanoscale and within the other two spatial dimensions that are allowed to exceed the nanoscale (Figure 1A); a 2D nanolaser could be a cylindrical nanowire, which confines light to the nanoscale in two spatial dimensions (Figure 1B); and a 3D nanolaser (Figure 1C) could be a nanosphere, in which light is confined in all three spatial directions to the nanoscale. These three nanolaser classes are known as the nanolaser dimensional prototypes [3].

**Figure 2:**
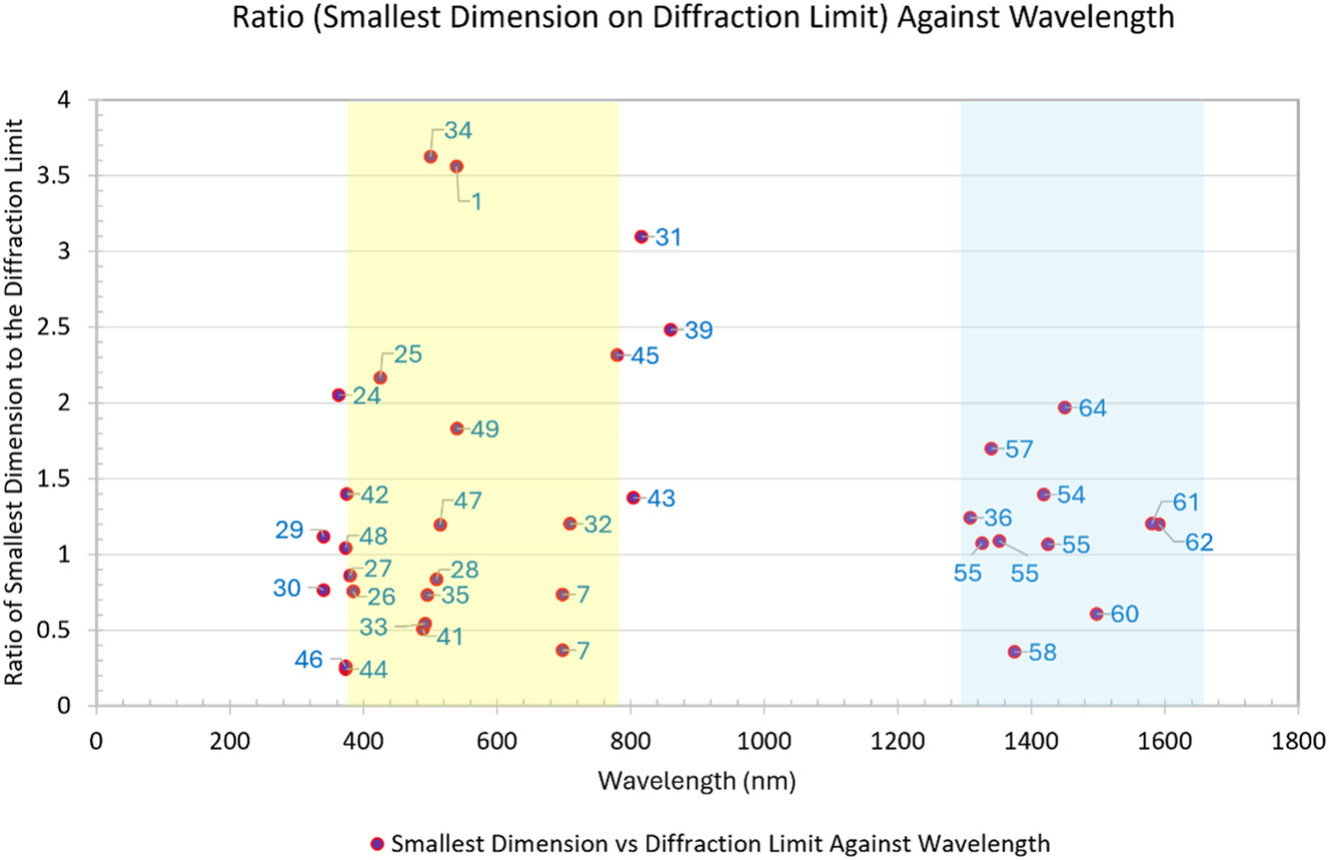
Comparison of the nanolasers reported in this review, plotted by the ratio of their smallest dimension to the diffraction limit against their emission wavelength. Yellow region – includes various types of nanolasers; blue region – includes only plasmonic nanolasers. The numbers indicate the respective reference.

Given this nanolasers definition, it is interesting to notice that not all the nanolasers we have considered in this review are also diffraction limited. Indeed, by plotting the ratio between the active medium smallest dimension and the corresponding diffraction limit, where the emission wavelength is considered, two clear regimes arise (Figure 2). The first regime (I – yellow) covers visible wavelengths from ∼380 nm to ∼780 nm. This regime is constituted by photonic, plasmonic, and hybrid plasmonic types of nanolasers. The second regime (II – blue) sits in the near infrared (NIR) spectrum region around the telecommunication wavelength, spanning from 1300 nm to 1650 nm. These nanolasers comprise solely of plasmonic lasers. It can be noticed that in the yellow region there is an equal distribution of above and below the diffraction limit nanolasers, whereas in the blue region the majority (>80 %) of the works we have considered are above the diffraction limit. This implies that most of the NIR nanolasers reported do not fully utilize the compression capabilities offered by plasmonic systems.

### 2.1 Photonic nanolasers

Photonic nanolasers are nanolasers that support solely photonic modes; hence they are fully diffraction limited. Consequently, the smallest volume a photonic nanolaser can achieve is approximately equal to ${\left(\lambda /2n\right)}^{3}$ , for a 3D photonic nanolaser.

**Figure 3:**
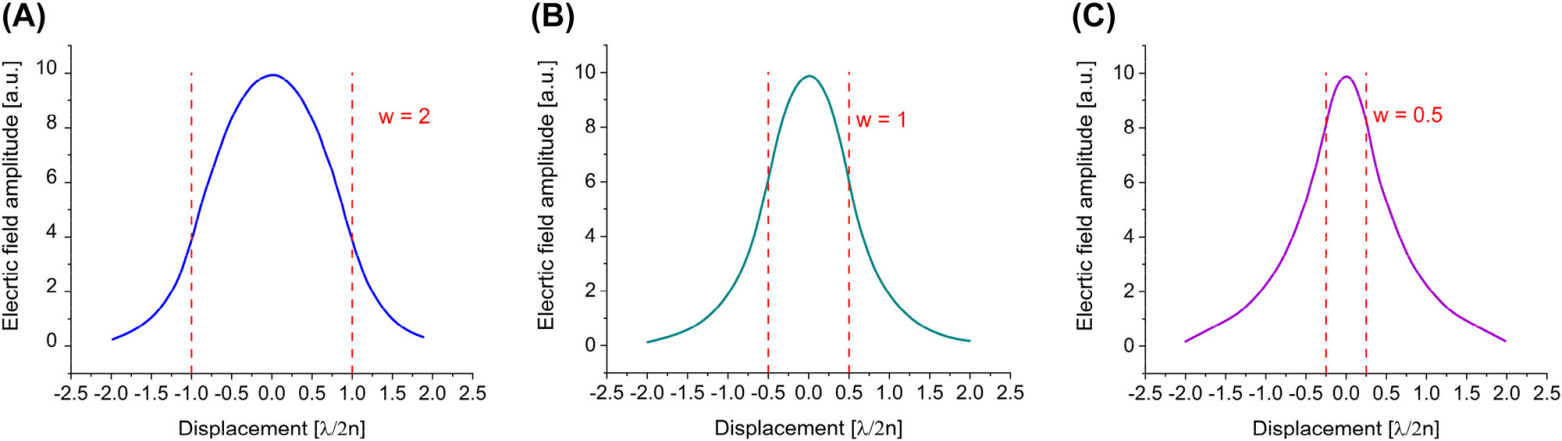
Electric field distribution of a photonic mode in an air-dielectric-air waveguide, where the mode profile distributions corresponding to each slab width overlapped with the physical size of the respective slab are plotted; (A) the width of the dielectric is $2\times \left(\lambda /2n\right)$ ; (B) the width of the dielectric is 1 × (*λ*/2*n*); and (C) the width of the dielectric is $0.5\times \left(\lambda /2n\right)$ .

Photonic nanolasers are mainly constituted by a dielectric waveguide which acts both as the active medium and the passive cavity, where light is reflected due to the high index contrast between the dielectric material and the cladding, which most of the time is air. These nanolasers are inherently leaky systems because much of the energy propagating within the dielectric is leaking outside the dielectric as evanescent field, hence not contributing to the overall lasing gain of the confined mode. This is shown in detail by the effective modal gain comparison between a photonic and plasmonic waveguide depicted in Figures 3 and 5. For instance, a 2D dielectric photonic waveguide slab that sits in air, when the width of the dielectric is larger than the wavelength, e.g. *w* = 2 × (*λ*/2*n*) (Figure 3A), the mode profile is primarily concentrated within the waveguide, although about 19.8 % of the field, in the plot of Figure 3A, is leaking out the waveguide as evanescent field, i.e. does not directly contribute to the effective modal gain [4]. As the width of the slab is reduced, the propagating mode is less confined within the waveguide, e.g. for *w* = 1 × (*λ*/2*n*) (Figure 3B) about 70 % is leaked outside the waveguide as evanescent field and for *w* = 0.5 × (*λ*/2*n*) (Figure 3C) this field is about 90 %. This inversely proportional leakage of the photonic mode for decreasing waveguide widths, approaching the diffraction limit, causes exponentially increasing thresholds.

Photonic nanolasers’ configuration, i.e. their cross-section, can be categorized to be what is called the IDA configuration, where “I” stands for insulator – usually a buffer layer, “D” for dielectric – usually the active medium as well as the cavity deposited on top of the insulator and “A” for air – usually the cladding, surrounding the structure. All nanolasers of the IDA configuration only support photonic modes. Currently only 2D and 1D photonic nanolasers have been demonstrated to date and include the photonic nanowire [5] and the photonic plane [6] nanolasers.

### 2.2 Plasmonic nanolasers/SPASERs

**Figure 4:**
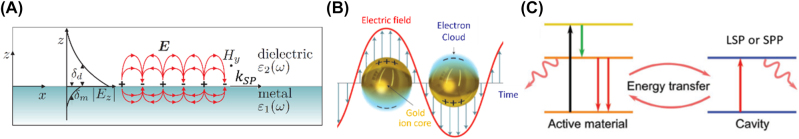
Plasmonic nanolaser fundamental physics. (A) SPP propagation along the surface of a metal; (B) LSP oscillation in the presence of an electric field; (C) three level energy diagram for stimulated emission in SPP and LSP process [3] reproduced with permission. [© 2019 WILEY-VCH Verlag GmbH & Co. KGaA, Weinheim].

Plasmonic nanolasers/SPASERs are a new breed of lasers that could allow light to be confined beyond the diffraction limit through coherent electron oscillations in metals. Although originally plasmonic nanolasers and SPASERs were considered different and still some confusion is found in the literature, their fundamental physics is the same. The only difference that can be highlighted is that SPASERs are utilizing localized surface plasmons (LSPs), Figure 4B [7], rather than surface plasmon polaritons (SPPs), Figure 4A [7], which are propagating waves in nature, at the dielectric-metal interface [8]. Nevertheless, given that SPASERs are truly nanoscale 3D confined nanolasers, we will treat them separately later in this review, even if the working principle of plasmonic nanolasers and SPASERs are interchangeable. This was first proposed by Bergman and Stockman in 2003, by realizing that surface plasmons could be amplified by stimulated emission [9]. The working principle is depicted in Figure 4C; an excitation of the gain medium, which can be by optical or electrical pumping, generates an inversion population between the two-level system, i.e. the laser transition energy levels, after a non-radiative decay into a metastable lasing level (represented by the green arrow in the figure).

These levels must be spectrally resonant (and the gain medium must also overlap the surface plasmon modal field) with the surface plasmon eigenmode in order to transfer the gained energy non-radiatively to the surface plasmon modes; in a conventional laser this energy is emitted either via spontaneous or stimulated emissions. Here the electrons can also be recombined by radiative decay (red vertical arrows) or non-radiative energy transfer to the surface plasmon mode. Once the energy has been transferred to the surface plasmon modes, a high local field is generated which in turn stimulate more emission from the gain medium either radiatively or into this surface plasmon mode: this is the feedback mechanism of a plasmonic nanolaser/SPASER [10].

It is now clear the fundamental difference between plasmonic nanolasers/SPASERs and conventional lasers: in the latter the feedback mechanism is supplied by an external passive resonator, or laser cavity, which requires to be at least half a wavelength in size along the propagation direction. On the contrary a plasmonic nanolaser/SPASER can be much smaller due to the fact that plasmonic modes can be of few nanometers in size [10].

Most of plasmonic nanolasers (i.e. those that use SPPs rather than LSPs) are waveguide-based and therefore they also utilize a Fabry–Perot cavity as a feedback mechanism, due to the reflection at the facets of the device, e.g. the facet of the gain medium which is intrinsically a photonic waveguide.

One of the great benefits of plasmonic cavities lays in their ability to confine light to sub-wavelength scales, where light confinement and light–matter interactions are greatly augmented [8].

**Figure 5:**
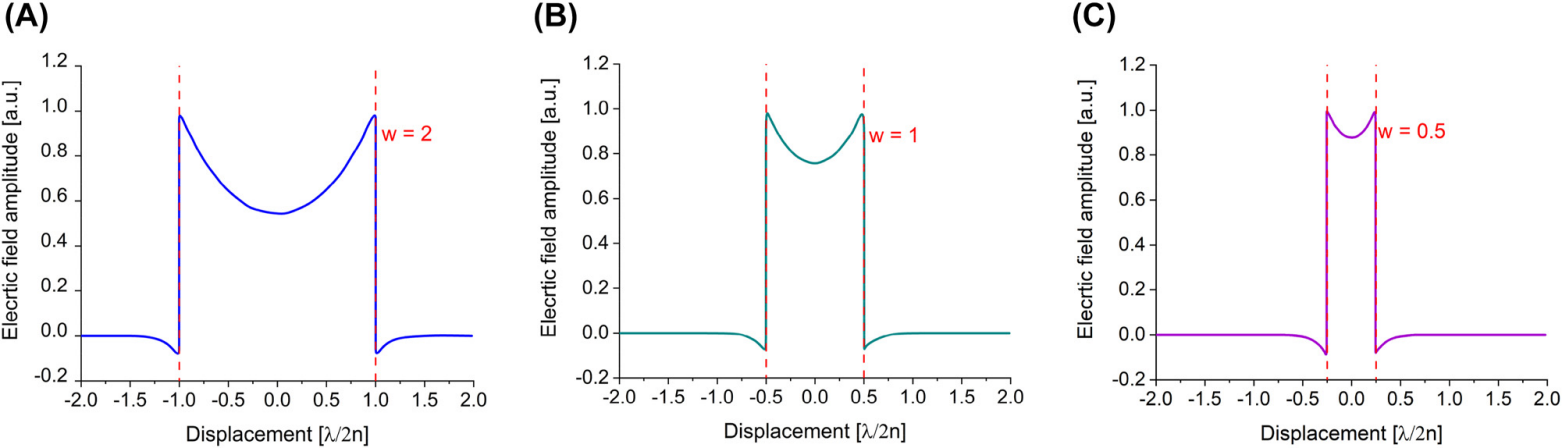
Electric field of a plasmonic mode in a metal–dielectric–metal waveguide, (A) with width *w* = 2; (B) with width *w* = 1 and (C) with width *w* = 0.5.

Consider again the insulator-dielectric-air (IDA configuration) waveguide slab discussed in Section 2.1; if the insulator is substituted by a metal film, then we obtain a plasmonic nanolaser. When this system is pumped, plasmonic modes, which are evanescent in nature, confine the electric field tightly within the active region of the dielectric; however, there is also a field decay into the metal which causes modal losses. Fortunately, the evanescent field, hence the confinement factor, exhibits a much longer decay length (larger confinement factor) in the dielectric than the one into the metal, by about two orders of magnitudes. Given that, the field penetrating in the dielectric, i.e. the gain medium, generates a net modal gain [8]. In the case where also the air is substituted by a metal, then the evanescent fields at both dielectric–metal interfaces interact with each other generating a highly confined field and a propagating mode, called gap plasmon mode [11]. Figure 5 shows how tightly confined the field profile distribution inside a metal–dielectric–metal (MDM) configuration is [4].

In case the dielectric is constituted by a gain medium, the light–matter interactions are enormously enhanced and the nanolasing process is promoted. However, although the light is tightly confined in the MDM slot, the field penetrating into both metals generates loss that must be taken into consideration. Indeed, as the width is reduced, more and more electric field is confined (with no cut-off, differently from what observed in Section 2.1 for photonic modes) and more and more field penetrates into the metal and is dissipated mainly by Ohmic losses [4]. MDM configurations show, for plasmonic nanolasers, the possibility of sub diffraction limited devices with low thresholds and nanoscale dimensions that do not exhibit the large leakage losses of small photonic nanolasers, but posses Ohimc losses.

As plasmonic nanolasers are reduced in size these losses become more critically significant and may inhibit lasing altogether. Reducing Ohmic losses in plasmonic materials is a highly active area of research in the miniaturization of plasmonic devices, both intrinsic and structural features of nanolasers may significantly impact the loss [12].

Plasmonic nanolasers can be broadly categorized by their materials configuration as reported by Li et al. [13]. These three main families are the MDM (metal–dielectric–metal), MDA (metal–dielectric–air), and MDHA (metal–dielectric–high index-air), depending on their field distribution as the “D” layer thickness *t*_D_ decreases. For the MDM configuration the electric energy remains strongly confined into the “D” layer with no cut-off, i.e. *t*_D_ → 0 [14]. In contrast, in the MDA configuration the electric energy shifts from the “D” to the “A” layer as *t*_D_ decreases, ultimately coinciding with a SPP configuration in air when *t*_D_ → 0. The MDHA configuration is intermediate between the MDM and the MDA, hence is generally called hybrid plasmonic. In this case, the energy remains strongly confined into the “D” layer, exhibiting less loss than a pure SPP configuration. As *t*_D_ → 0, the electric energy ultimately resides in the “H” and “A” layers. Only the MDM can reach a deep-subdiffraction limit confinement, whereas the MDHA and the MDA are limited to the moderate- and near-subdiffraction limit confinements, respectively.

**Figure 6:**
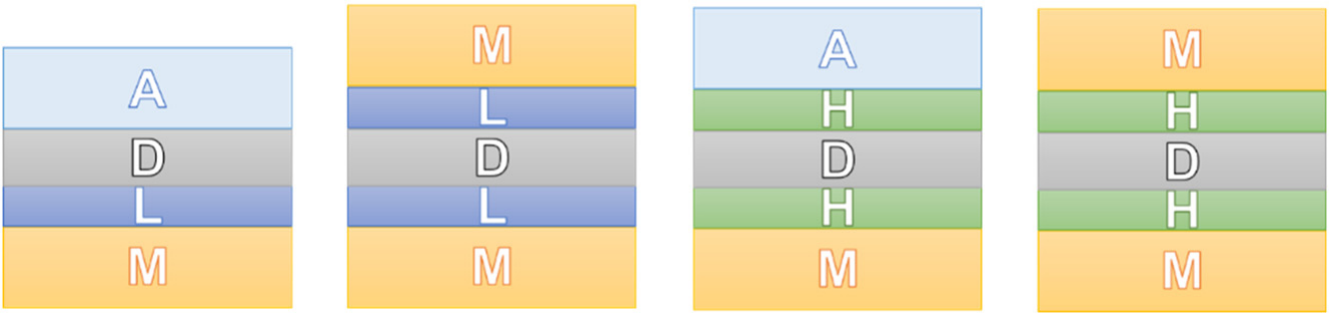
Plasmonic nanolaser configurations. M: metal; L: low index medium; D: dielectric medium, the active medium; H: high index medium.

Given that the gain medium “D” is in contact with the metal “M”, quenching of spontaneous emission happens. This can be removed by inserting a low index buffer layer “L” between the metal and the gain medium. Therefore, the MDM and the MDA families become the MLDLM and the MLDA, respectively, which are often refers in the literature as MISIM and MISA [15] where the “I” layer corresponds to the “L” layer, in our case, and the “S” layer is the gain medium (i.e. the semiconductor) corresponding to the “D” layer in our case. The low-index buffer layer insertion makes the MLDA remain in the near-subdiffraction region, whereas the MLDLM can no longer reach the deep-subdiffraction region, because the electric field is drawn to the low-index buffer layer “L”. Li et al. [13] showed that by simply substituting the “L” layer with a high-index medium “H”, the corresponding nanolaser performs better than its low-index counterpart because the field is now pushed into the "D" layer where it can consequently minimise the gain threshold. For instance, an MHDHA is preferred over MLDHA as well as the MHDHM is better than the MLDLM. These structures are displayed in Figure 6.

The MHDHA and MHDHM configurations replace the low index dielectric insulator material seen in the MIDA and MIDIM structures with a high index dielectric material (usually a semiconductor) and are both currently theoretical options that will be examined in more detail in Section 3.4.

### 2.3 Properties of nanolasers

The general method for determining the performance of nanolasers lays in the analysis of the Purcell factor [16], the *β* factor [17], the quality factor, the lasing threshold, and the operating temperature.

The Purcell factor for a laser represents the ratio between the spontaneous decay rate of a radiative mode from an emitter in a resonator compared to the radiative mode of the same emitters in free space; it in essence represents the enhancement or inhibition of the spontaneous emission rate of an emitter in a cavity compared to emission in free space. If the radiative mode is the lasing mode of the cavity, a high Purcell factor is desirable as it represents an increase in the coupling between photons spontaneously emitted and the lasing cavity mode; consequently, increasing the rate of stimulated emission in the cavity, lowering the gain threshold and therefore augmenting the overall lasing efficiency. The Purcell factor for a cavity is defined as:

(1) $$F=\frac{3}{4\pi^{2}}{\left(\frac{\lambda_{0}}{n}\right)}^{3}\frac{Q}{V_{\text{m}}}$$

where *F* is the Purcell factor, *λ*_0_/*n* is the effective wavelength (*λ*_0_ is the wavelength in vacuum, *n* is the refractive index of the gain medium), *Q* is the cavity quality factor, i.e. directly proportional to the energy stored in the cavity (hence the time photons are stored in the cavity), and *V*_m_ is the mode volume, i.e. the spatial extent of the modal field within the cavity. To increase the Purcell factor, *Q* must be maximized and the mode volume minimized.

The quality factor *Q* represents the ratio between the energy stored and the energy lost in one oscillation cycle of the cavity mode. In both photonics and plasmonics cavities the quality factor may be affected by the material, the quality of the reflectors, the accuracy to which the nanostructure is made, and the surface roughness of the cavity. As a result, fabrication has become a highly active area of interest to improve the loss and therefore increase the *Q* factor.

The *β* factor, also known as the spontaneous emission coupling factor, represents the fraction of spontaneous emission that is coupled into the lasing mode of the cavity compared to all other spontaneous emission modes. The *β* factor is defined as:

(2) $$\beta =\frac{F^{\left(1\right)}}{\underset{i}{\sum }F^{i}}$$

The higher the *β* factor of a cavity, the higher the rate of spontaneous emission coupled into the lasing mode. When *β* = 1, all spontaneous emissions in the laser cavity are coupled into the lasing mode greatly increasing the stimulated emission in the cavity. A cavity with a *β* factor of one is known as a thresholdless cavity in which pumping can immediately produce lasing with little to no energy required. This is one of the ultimate goals of nanolaser design.

The laser threshold represents the point at which the gain supersedes the losses in a cavity. Increasing the *β* (towards one) and Purcell factors acts toward decreasing this threshold.

**Figure 7:**
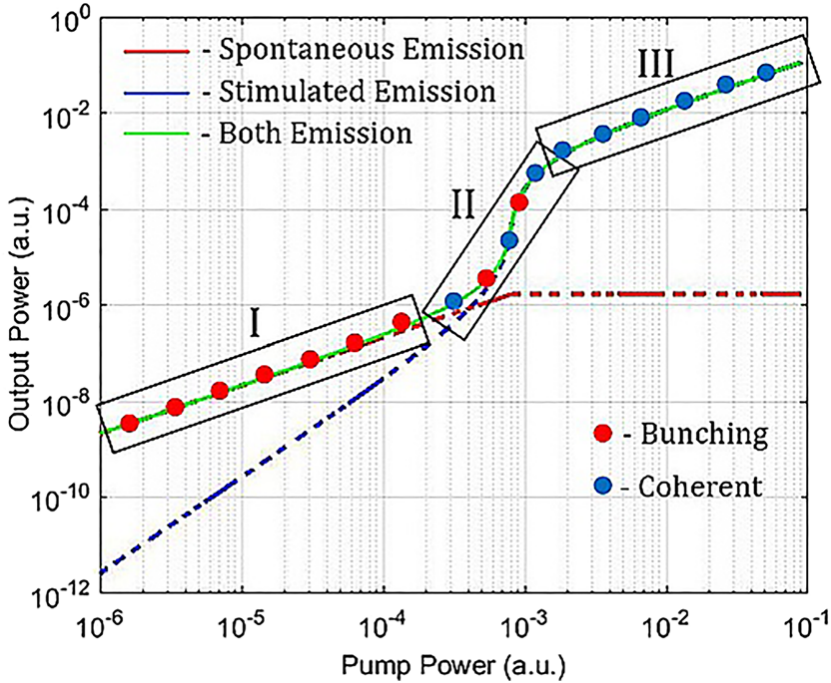
Characteristic *S* shape on a log-log distribution of laser output power and pump power [18].

Additionally, it is important to keep in mind that the photon statistics in a laser light is completely different from other incoherent light sources. This is well represented by the second correlation function and the plot in Figure 7, where the Poissonian statistic distribution of photons in a laser light clearly appears as soon the stimulated emission takes over the spontaneous emission going from bunched photons and super-Poissonian photon statistics (region I) to anti-bunched photons governed by a Poissonian statistics (region III), transiting from a region mixed with the two regimes (region II).

Therefore, region I exhibits a second order correlation function larger than 1, i.e. a super-Poissonian photon statistical distribution, and region III exhibits a second order correlation function equal to 1, i.e. a Poissonian photon statistical distribution. This also implies that when *β* = 1, the plot in Figure 7 will appear essentially without region I and II and hence the statistical distribution of the photons is Poissonian, which is a reflection of a pure classically coherent light.

The final figure of merit concerns the nanolaser operation temperature; room-temperature operation is desirable since it forms the backbone of many applications and reduces the overall cost and complexity. Laser design has a large impact over the operation temperature of nanolasers and it has been shown that *β* is highly temperature dependent. Some designs yield high *β* factors in the lasing mode at cryogenic temperatures, which degrades with increasing temperature, while others have a high *β* factor closer to room temperature, which degrades with decreasing temperature [19].

It is fundamental in the design of nanolaser cavities that all the properties listed above are considered. Gu [20] summarizes this well in the following list of design aims to maximize the spontaneous emission efficiency in the gain medium:

1. Eliminate, as far as possible, the wasteful resonant modes from the spectral region of the gain.
2. Minimize, as far as possible, leaky modes into free space.
3. Utilize the resonant mode with a sufficiently high cavity *Q* factor, and even more importantly, an as small as possible mode volume, in order to maximize the interaction between the optical mode and the gain medium.
4. Align, as much as possible, the gain spectrum to the resonant mode in (3).

#### 2.3.1 Small cavity volumes

**Figure 8:**
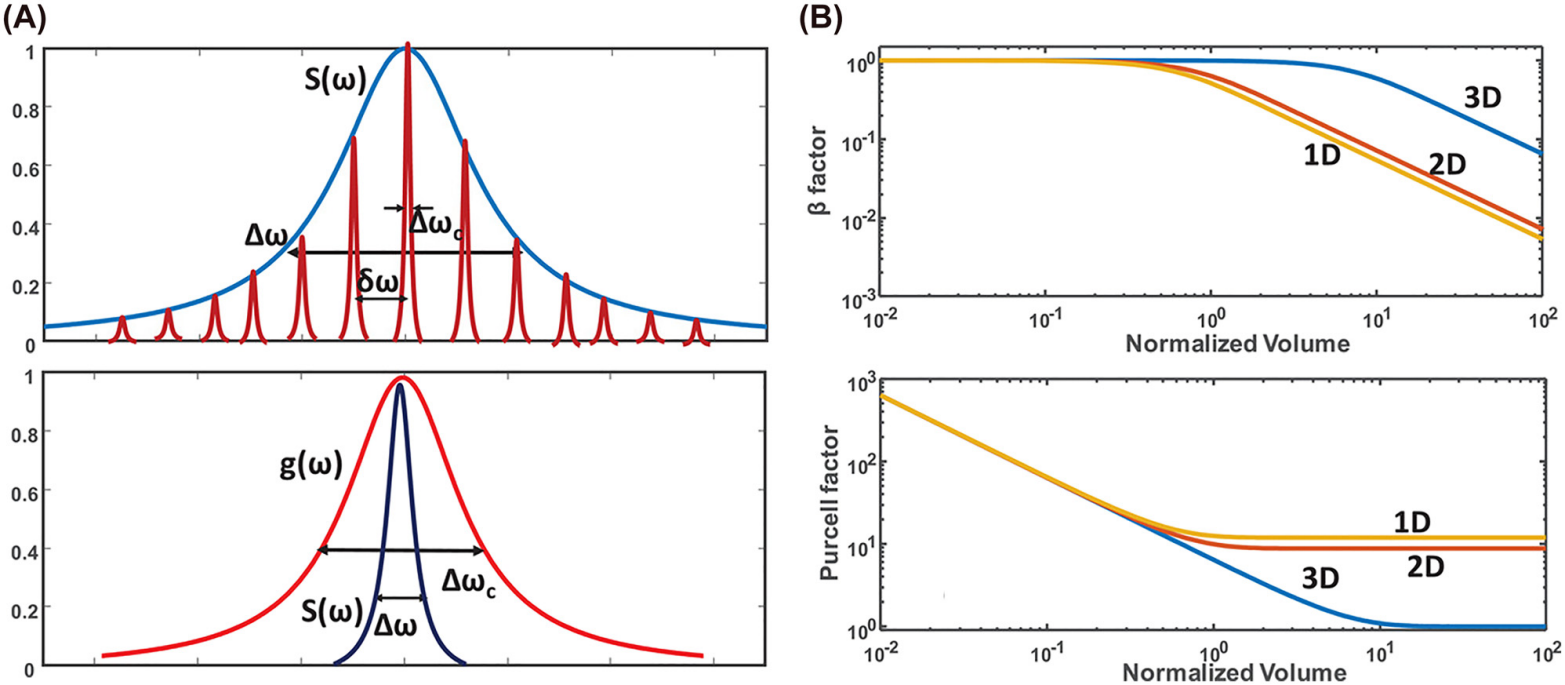
Multimode oscillations and *β* factor in plasmonic nanolasers. (A) Top: overlap of the gain curve (blue) with the lasing modes (red), bottom: overlap of the gain curve (blue) with the wide lasing mode (red) [21] reproduced with permission; (B) top: comparison of 1D, 2D, and 3D cavity *β* factors against volume, bottom: comparison of 1D, 2D, and 3D Purcell factors against volume [21] reproduced with permission. [© 1999–2023 John Wiley & Sons, Inc].

As the volume decreases, the number of cavity modes decreases. Multimode lasing oscillations reduces *β* and increases laser threshold (Figure 8A top panel). As the volume is decreased towards or below the diffraction limit, the gain curve shrinks towards its mean value, which increases the number of electrons that can be coupled to the lasing mode, hence augmenting the *β* factor towards unity and decreasing the threshold (Figure 8A bottom panel). When plasmonic nanolasers are considered, the mode volumes within the cavity become increasing small, which in turn increases the Purcell factor lowering the lasing threshold. The combined effect of the decreased mode volume on the *β* factor and Purcell factor is summarized in Figure 8B for 1D, 2D, and 3D nanolaser structures. This description of the effect of cavity volume on nanolaser properties is generalized and does not consider losses, both the effect of volume and losses must be considered in the study of nanolasers.

#### 2.3.2 Fast dynamics

Another consequence of the small cavity and respective mode volume is an increase in the modulation speed of nanolasers. As the mode volume is decreased the Purcell factor increases. It has been shown that the modulation speed in laser cavities is directly linked to the Purcell factor [21]. Fast modulation speeds provide exciting possibilities for the applications of nanolasers for high-speed data distribution and are discussed in Section 6.

#### 2.3.3 Coherence

Nanolasers differ from conventional lasers in their coherence properties. The reduced number of allowable modes in nanolaser cavities increases the Purcell and *β* factors increasing coupling to the lasing mode which acts to increase the temporal coherence. The resulting small linewidth for these devices could enhance data processing. Compared to macroscopic lasers, nanolasers suffer from poor spatial coherence because emitted light diffracts like a point source. Collecting and guiding the emitted radiation is a crucial problem that must be addressed for the many possible applications of nanolasers. Arrays of nanolasers have been shown to achieve spatial coherence and are discussed in Section 4.

#### 2.3.4 Low lasing threshold

Nanolasers tend to have low lasing thresholds due to the low mode volume, high *β* factor, and high Purcell factor. The main challenge in achieving low thresholds in nanolasers is losses, and as such loss mitigation, especially metallic losses in plasmonic nanolasers should be a major focus in nanolaser development.

#### 2.3.5 Wavelength tunability

Modulation of the wavelength of nanolasers either dynamically or chemically through band gap engineering creates the opportunity for a wide range of novel and enhanced applications as well as manufacturing processes that produce a wide range of emission frequencies. To date nanowires have been shown to operate from the UV to telecommunications wavelength range [22]. Most nanolaser active regions are composed of semiconductors which allow easy modification of the chemical composition and subsequent emission. Dynamic tuning is still in its adolescence and remains an active research problem that may revolutionize the operation of nanolaser devices.

## 3 Nanolasers and their properties

Here the definition provided in Section 2 is utilized to provide an overview and characterization of the current and reported state-of-the-art of nanolaser research based on the nanolaser configuration type and sub-grouped into their dimensional confinement category. The configurations outlined in Section 2 provides the bedrock for this analysis and resultingly the IDA, MLDA, MLDLM, MHDHA, MHDHM, and novel configurations are analyzed, where “L” stands for insulator material with a low refractive index subject to photonic or plasmonic configurations respectively, “D” for Dielectric which is the active medium, “A” is Air, “M” stands for Metal, and “H” for a High refractive index material. The mode of operation, properties, and limitations are discussed.

### 3.1 IDA nanolasers

The IDA configuration for nanolasers supports solely photonic modes within their cavities which significantly inhibit their lasing capabilities approaching the diffraction limit; it is basically a photonic waveguide for a Fabry–Perot geometry or a photonic ring resonator for a circular cavity geometry. Due to this physical constraint only 1D and 2D IDA nanolaser configurations have been demonstrated to date, since 3D IDA nanolasers would be required to lase in the deep UV to satisfy the nanolaser definition which has not yet been achieved for any nanolaser. 3D perovskite nanocuboid lasers have been reported to approach the defined nanolaser dimension but still sit outside the criteria limit of less than 400 nm in one dimension set out in Section 2 [1].

#### 3.1.1 1D IDA configuration nanolasers

The IDA nanolaser type comprises of two subsets, the square/film type nanolasers and the photonic microdisk lasers, which in some work has one dimension within the nanoscale making these 1D nanolasers. For the square/film type nanolasers both edge emission and vertical emission have been demonstrated.

##### 3.1.1.1 Square and film type photonic nanolasers

**Figure 9:**
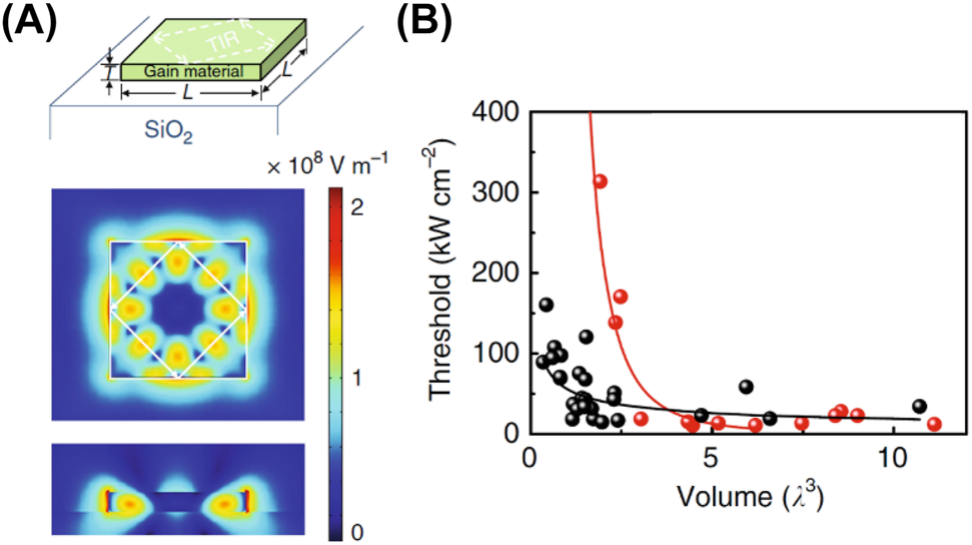
Nanosquare laser example. (A) Top: IDA structure of the nanosquare laser; middle: top-down view of the mode profile; bottom: side profile of the mode profile [6]; (B) comparison of photonic (red) versus plasmonic (black) nanosquare lasing threshold, thickness 100 < *T* < 150 nm [6] licensed under Creative Commons Attribution 4.0 International License.

Currently, the only 1D IDA nanolaser structure that has been demonstrated is the photonic square nanolaser constituted by a CdSe semiconductor crystal gown on an insulator [6] (top panel of Figure 9A). Photonic square nanolasers have microscale widths and nanoscale thicknesses with modes determined by total internal reflections between the edges of the structure [23]. Figure 9A depicts the general structure of these devices (top), and the electric field magnitude of the resonat modes (bottom), respectively along the width and the thickenss cross-sections, for a nanosquare of side length *L* = 700 nm and thickness *T* = 100 nm [6]. The electric field magnitude (bottom graphs of Figure 9A) clearly shows the field being totally internally reflected between the edges, strong interaction with the active medium, and the high leakage to the environment. Figure 9B compares the lasing threshold of a room-temperature CdSe nanosquare in the IDA configuration (red) with the plasmonic MIDA (black) configuration for crystal thicknesses between 100 and 150 nm [6]. For large thicknesses, the photonic nanolaser shows little change in the lasing threshold however, as the diffraction limit is approached a rapid increase in the lasing threshold is observed due to the high leakage of the field. This threshold limits the viability of the photonic square nanolaser at sizes approaching the diffraction limit and little improvement in performance should be expected.

#### 3.1.2 2D IDA nanolasers

The 2D IDA configuration is dominated by the photonic nanowire laser, in which a single straight crystalline semiconductor gain medium is grown on an oxide substrate. These nanolasers are hundreds of nanometers to microns in length and between tens to a few hundred nanometers in diameter with varying cross-sectional geometries.

**Figure 10:**
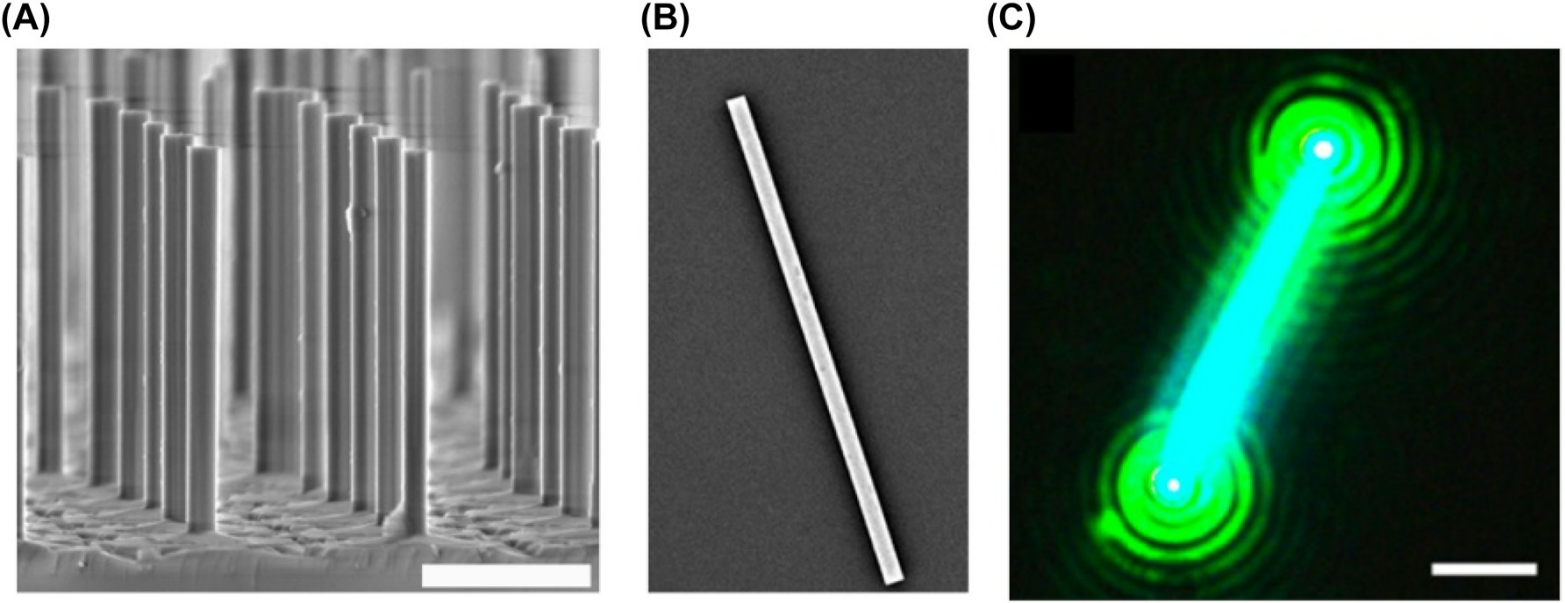
IDA nanolaser examples. (A) SEM images of GaN nanowire columns [25] reproduced with permission [Optica Publishing Group]; (B) SEM image of a single CsPbBr_3_ nanowire on quartz [26] reproduced with permission [PNAS]; (C) lasing excitation of CsPbBr_3_ nanolaser [26] reproduced with permission [PNAS].

The high refractive index of the semiconductor compared to the surrounding environment confines light within the active medium and the end facets form a Fabry–Perot cavity that supports photonic modes. Once threshold is achieved, lasing occurs directionally along the length of the nanowire escaping through the end facets. Figure 10C, beautifully shows the nanowire lasing once the threshold is overcome. The first nanowire lasers were demonstrated by Huang et al. [24] using ZnO crystals grown vertically on a sapphire substrate with lasing observed in the UV. These nanowires are intrinsically waveguides, form a cavity due to the crystalline edge facets ( i.e. a Fabry–Perot cavity when the refractive index of the surrounding is lower than the one of the crystals, e.g. air), exhibit fast modulation speed, and their composition can be easily chemically controlled. Since the first demonstration of photonic nanowire lasers, an abundance of demonstrations have been reported with a variety of gain materials, emission wavelengths, geometries, and operating conditions, some of which are shown in Table 1.

**Table 1:** Other photonic nanowire laser demonstrations including gain material composition, emission wavelength, nanolaser diameter, lasing conditions including temperature of operation and pump type. (RT = room temperature, CW = continuous wave, MALH = methylammonium lead halide, CLH = cesium lead halide).

Year	Gain medium	λ (nm)	*D* (nm)	Lasing conditions	References
2001	ZnO	380–385	20–200	Pulsed (RT)	[24], [27]
2003	CdS	490–495	80–200	Optical (8 K)	[28]
		Electrical (8 K)			
2004	ZnS	340	80–100	Pulsed (RT)	[29]
2005	ZnCdS	340–390	50–80	Pulsed (RT)	[30]
		485–515			
2009	GaAs	816	200–500	Pulsed (4.2 K)	[31]
2011	CdSe	709	150–350	Pulsed (RT)	[32]
2012	GaN	363	135–145	Pulsed (RT)	[25]
2013	CdS	493	250	CW (120 K)	[33]
2015	MALH	500–790	300	Pulsed (RT)	[34]
2016	CLH	425–545	200–2300	Pulsed & CW (RT)	[26]

**Figure 11:**
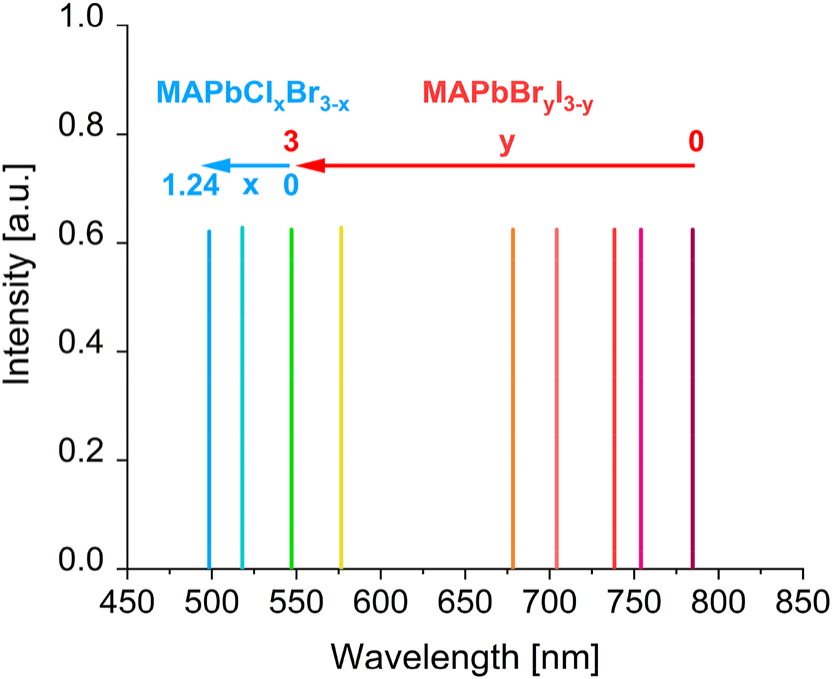
Wavelength range of methylammonium lead perovskite through the change of the halide composition.

The 2015 methylammonium lead halide and 2016 cesium lead halide nanowire lasers [26], [34] exhibited wavelength tunability through the control of the halide concentration during the nanowire fabrication process with lasing throughout most of the visible spectrum, providing possible opportunities to create multicolored communications and displays; Figure 11 shows the emission peaks as a function of the halide composition for methylammonium lead halide [34]. It is clear from Table 1 that most of these nanolasers lie on the borderline of the 2D nanolaser definition outlined in Section 2 and are affected by the photonic mode leakage problem when approaching the diffraction limit. Nevertheless, the combination of natural light guiding, wavelength tunability, room temperature operation, and fast modulation speed make photonic nanowires a good candidate for direct integration in optical circuitry, high speed data processes, and multiwavelength signals generation, even if further miniaturization is unlikely to progress into the strict nanoscale regime.

##### 3.1.2.1 MLDA nanolasers

The devices presented in this section support either hybrid plasmonic-photonic modes or solely plasmonic modes only in case “L” is absent. However, in most cases, a low index insulating buffer layer “L” is necessary to avoid quenching the emission from “D” in close contact with the metal “M”. Therefore, we could state that the MLDA nanolasers are mainly based on a hybrid plasmonic mode, i.e. a hybridization between a pure photonic mode (in “D”) and a SPP mode (in “ML”). This section will outline the historical and current developments of the MLDA configuration again using our nanolaser definition to more clearly subcategorize these nanolasers. No 3D MLDA nanolaser has been successfully demonstrated to date.

#### 3.1.3 1D MLDA nanolasers

Under the category of 1D MLDA nanolasers two major geometries were reported, the plasmonic nanosquare, the analog of the photonic nanosquare, and the nanodisk nanopan laser.

##### 3.1.3.1 Plasmonic nanosquare laser

**Figure 12:**
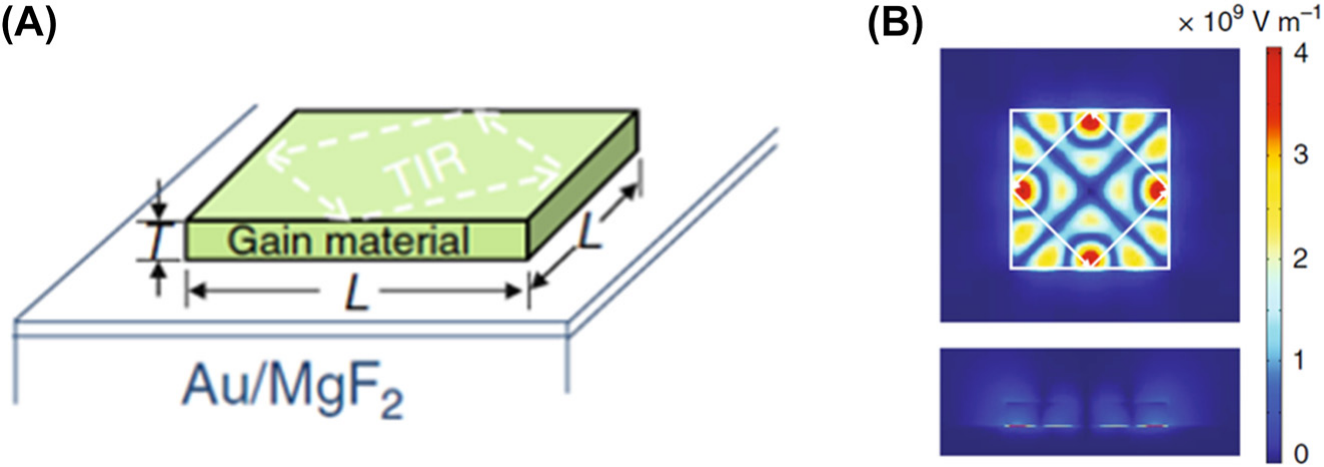
Plasmonic nanosquare example. (A) Plasmonic nanosquare schematic; (B) side and top view of the electric field intensity of within the nanosquare laser [6]. Licensed under Creative Commons Attribution 4.0 International License.

Ma et al. [35] first showed plasmonic lasing in nanosquares implementing a CdS nanosquare active medium on silver, which is schematically represented in Figure 12A. Photonic modes in the nanosquare hybridize with plasmonic modes at the metal-dielectric interface tightly confining light in the insulator region, Figure 12B shows the tightly confined electric field profile. This geometry promotes total internal reflection at the side walls of the gain material in the way a cavity mode is created, which in turns acts as the feedback mechanism for the nanolaser. The ultra-small mode confinement (∼45 nm) allows this nanosquare structure to fully reach the nanoscale. Furthermore, the hybrid plasmonic mode was shown to increase the spontaneous emission rate of the of the gain medium’s lasing transition by 18-fold, drastically reducing the lasing threshold compared to the pure photonic nanosquares.

Indeed, a comparison of photonic and plasmonic nanosquare nanolasers shows that plasmonic nanosquare lasers exhibit far lower thresholds than their photonic counterparts [6]. Thresholds in 1D photonic nanolasers are generally lower than in plasmonic ones for large thicknesses due to low mode leakage, however as the diffraction limit is approached, photonic modes become less confined, while plasmonic modes remain highly confined. Nevertheless, the plasmonic lasers threshold increases as Ohmic losses rise for very small thicknesses. This is shown in Figure 9B for CdS nanosquares on gold. The small mode confinement, natural 2D waveguide shape, room temperature operation and large light matter interactions at small thicknesses supplies compact devices that may have applications in optical circuitry and high-volume data systems.

##### 3.1.3.2 Nanodisk nanopan lasers

**Figure 13:**
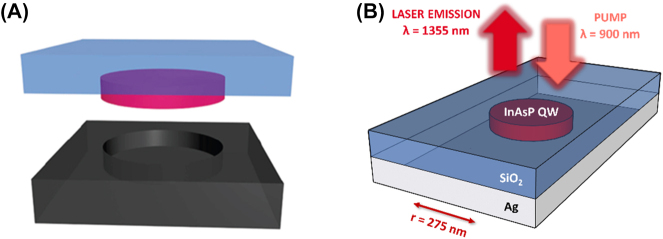
Nanodisk nanopan lasers examples. (A) Nanodisk nanopan separated schematic [36]. Reproduced with permission. [ACS NanoLetters]; (B) nanodisk nanopan integrated schematic.

Nanodisk-nanopan lasers, first demonstrated in 2009 [36], are generally constructed as a planar semiconductor gain medium which sits in a metal cavity (pan) and support whispering gallery plasmonic modes at the metal-dielectric interfaces, in the case of nanodisks, or hybrid plasmonic modes, in case of nanowires. The first published demonstration of the former consisted of an InP nanodisk embedded with four planar InAsP quantum wells with micrometer scale diameters and thicknesses of ∼200 nm, grown on glass and covered in silver (Figure 13B).

This structure supports both plasmonic SPP modes and optical modes at cryogenic temperatures, however it is reported that an ultra-confined TM SPP mode at the metal dielectric interface (bottom of the pan) was responsible for lasing [37]; for diameters less than ∼1500 nm the optical modes are severely degraded. This mode is tightly localized at the bottom-side metal-dielectric interface with an ultrasmall mode volume of $0.56{\left(\lambda /2n\right)}^{3}$ , far below the diffraction limit. However, it was found that the plasmonic mode exhibits a significant decrease in *Q* factor for larger temperatures, which in turn increases the threshold preventing lasing at room temperature. Nanodisk–nanopan lasers have also theoretically been shown to be highly sensitive to surface roughness with plasmonic *Q* factors severely degrading for an increase in average roughness of 1 nm, highlighting the need for ultrasmooth fabrication techniques in their construction [38]. Another study compared the lasing threshold difference between metal coated, insulator separated circular, square, and hexagonal nanodisk–nanopan structures and their noncoated analogue with thicknesses of ∼300 nm grown on Silicon [39]. This study revealed that laser thresholds were lower in the purely photonic structures with diameters above the diffraction limit; however, the plasmonic structures allowed lasing below the diffraction limit. Furthermore, it was found that the plasmonic lasers operated at far lower temperatures than their photonic counterparts with the metal acting as a heat sink for the semiconductor.

Overall nanopan–nanodisk lasers are highly successful in confining light beyond the diffraction limit with higher thresholds than their photonic counterparts but with smaller geometries and better thermal properties, moreover, their structure may permit electrical injection, although this has not yet been demonstrated. This would allow for their integration with optoelectronics and would be a step towards purely optical circuitry. Reducing Ohmic losses, which degrade the plasmonic modes, and optimizing the nanoscale and microscale geometries are likely to improve the performance of these devices. Further investigations on the effect of insulators on these structures should be conducted to examine their effect on Ohmic losses at higher temperatures.

#### 3.1.4 2D MLDA nanolasers

**Figure 14:**
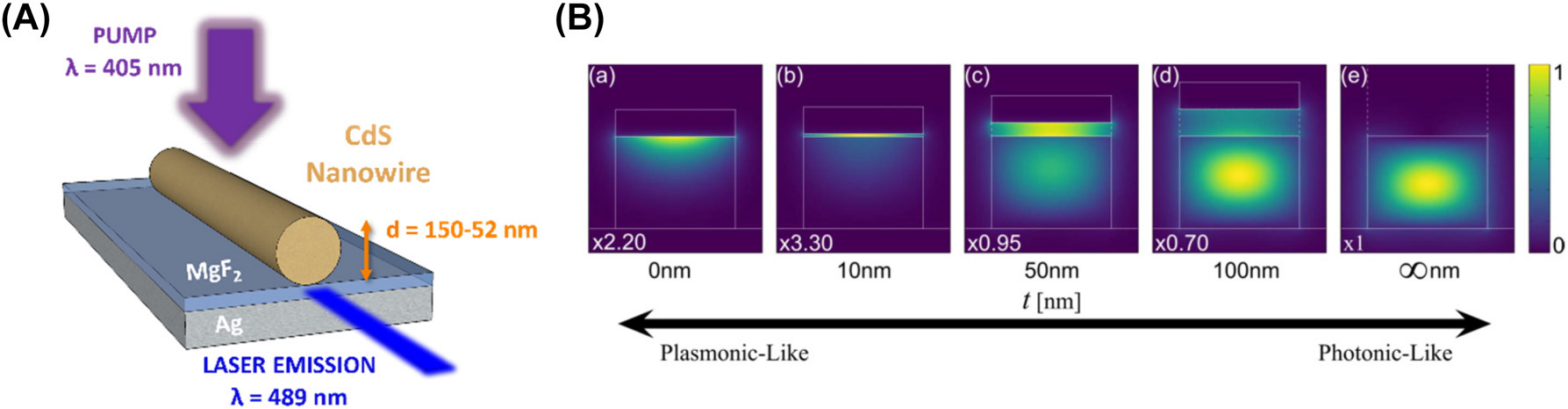
2D MLDA nanowire example. (A) Schematic of the CdS plasmonic nanowire laser; (B) fundamental mode of a hybrid plasmonic architecture for buffer layer thicknesses *t* of (a) *t* = 0 nm, (b) *t* = 10 nm, (c) *t* = 50 nm, (d) *t* = 100 nm, and (e) *t* →∞. Each subfigure plots the magnitude of the Poynting vector normalized to its total power and multiplied by a scaling factor (bottom left) to equate maximum intensity. The constitutive materials’ linear refractive indices are SiO_2_ as buffer (*n* = 1.44), Si (*n* = 3.47), air (*n* = 1.00) and Au (*n* = 0.52) at telecom wavelength [40]. Reproduced with permission. [Optica Publishing Group].

Plasmonic nanowire lasers dominate the 2D MLDA configuration and consist of a nanowire on a metal usually separated by a low index spacer, i.e. a buffer layer. These nanolasers differ from photonic nanowire lasers as they can support hybrid plasmonic-photonic modes, purely photonic modes and even solely plasmonic modes as a function of the thickness of the buffer layer as shown in Figure 14B [40]. These modes are tightly bound within the low index spacer and as a result allow for deep-subwavelength light confinement.

One of the three initial plasmonic lasers demonstrated in 2009 consisted of a CdS cylindrical nanowire grown on a flat silver layer separated by a low refractive index spacer (MgF_2_) [41] as shown in Figure 14A. The team not only demonstrated lasing in the plasmonic nanowires, for diameters between 52 and 150 nm, but also compared their lasing threshold with photonic CdS nanowire lasers for a range of diameters. The photonic laser was shown not to lase under a diameter of approximately 140 nm while the plasmonic nanolaser produced sub-diffraction limited lasing for diameters down to 52 nm. This behavior is explained by the mode leakage in the photonic laser and the hybrid plasmonic modes strongly confining light into the insulator region in subwavelength volumes for the plasmonic nanolaser.

Figure 14B shows the electric field profile of the hybrid plasmonic mode exhibiting tight confinement in the insulator region, i.e. the buffer layer [40]. This hybrid plasmonic mode area was found to be as small as $0.01{\left(\lambda /2\right)}^{2}$ which is far beyond the diffraction limit [41]. The high *β* factor, low threshold, and fast dynamics of this laser showed promise in the new nanolaser design [41].

In Table 2 we have summarized a selection of reported plasmonic nanolasers alongside their main operative conditions.

**Table 2:** Other photonic nanowire laser demonstrations including gain material composition, emission wavelength, nanolaser diameter, lasing conditions including temperature of operation and pump type. (RT = room temperature, CW = continuous wave, MALH = methylammonium lead halide, CLB = cesium lead Bromide).

Year	Geometry	Gain medium	*λ* (nm)	*D* (nm)	Lasing conditions	References
2009	Cylindrical	CdS	489	50–400	Pulsed (cryogenic)	[41]
2014	Triangular	GaN	370	50–300	Pulsed (RT)	[42]
2015	Hexagonal	GaAs/AlGaAs	804	150	Pulsed (125 K)	[43]
2015	Hexagonal	ZnO	373	28	Pulsed (RT)	[44]
2016	Rectangular	MALH	768–796	200–500	Pulsed (RT)	[45]
2016	Hexagonal	ZnO	372–386	30	Pulsed (77–353 K)	[46]
2018	Rectangular	CLB	533	120	Pulsed (RT)	[47]
2018	Rectangular	ZnO	376.7–382		Pulsed (147–330 K)	[48]
2020	Rectangular	MALH	540–555	238	Pulsed (RT)	[49]

**Figure 15:**
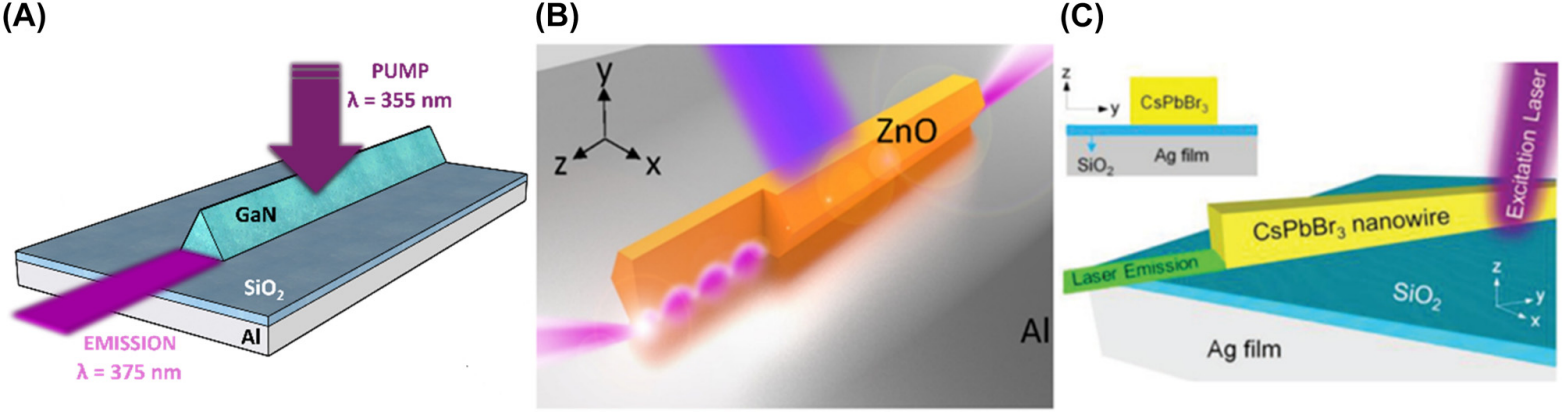
Schematics of lasing in (A) a triangular GaN plasmonic nanowire geometry; (B) a hexagonal ZnO plasmonic nanowire geometry [46] [© 2016 American Chemical Society]; (C) a rectangular cesium lead bromide plasmonic nanowire laser geometry [47] [© 1999–2023 John Wiley & Sons, Inc.].

Although these nanolasers can operate below the diffraction limit, they tend to have higher thresholds than their photonic counterparts, on the order of MW/cm^2^ compared to those listed in Table 1, which require a pump of the order of kW/cm^2^. This is due to Ohmic losses and scattering losses of the plasmonic modes at the metal–dielectric interface. Great effort has been made to reduce losses in plasmonic nanowire lasers by changing the geometry and composition of the gain materials of these devices, however no great progress has been made to date. Some of these solutions include changing the spacer thickness [50], using different gain medium cross-sections such as triangular [42], rectangular [47], and hexagonal [46] as shown in Figure 15, respectively. These changes aim to better confine light at the metal–dielectric interface to reduce the threshold. These high thresholds must be reduced to make these light sources commercially viable.

Despite the higher thresholds, these devices very successfully confine light well below the diffraction limit reducing the number of modes that the nanowire can support, subsequently increasing the *β* and Purcell factors. They not only have all the properties that photonic nanowires possess, including directional output and wavelength tunability by means of band gap engineering, but also exhibit superior modulation speeds and the intrinsic possibility of electrical injection, although not yet demonstrated [20]. The fast modulation speed is owing to the small mode volumes and the high Purcell factor. Thus far nanowire pulses were recorded at 800 fs in a ZnO nanowire laser [51] and at 1.2 ps in a methylammonium lead bromide nanowire laser [49], both at room temperature. This fast modulation speed is highly exciting for high-speed data application in on chip integration of nanowires.

Plasmonic nanowires are highly successful in confining light to sub-diffraction limited scales and are only inhibited by the large metal losses. Their versatility in wavelength, fast modulation speed, natural waveguided shape, and very compact size, could lend applications in storing and transmitting data in on-chip applications, as well as supplying the appropriate light sources for lab-on-chip devices, specifically addressing the biomedical mobile sensing sector. With the potential for electrical injection, these devices may eventually realize the ultimate nanolaser for very compact, rugged and therefore portable applications. Research should clearly aim to further reduce the lasing threshold, attempting to use *n* and *p* junction to incorporate electrical injection, and formulate designs to effectively couple these nanolasers with other plasmonic devices. Recent studies have started this work with a nanolaser in a V-shaped groove showing output coupling to the propagation modes of the groove [52]. It has to be noted that plasmonic nanorods are plasmonic nanowires with lengths on the order of a few wavelengths and approach full 3D nanolaser confinement; however these nanolasers still sit just outside the 3D confinement outlined in the definition section of work. One study on lasing in GaN nanorods presented an average length of just 480 nm in length and 30 nm cross section opening a window for deep sub-wavelength lasing [53].

### 3.2 MLDLM configuration nanolasers

The MLDLM configuration is constituted by a second major class of plasmonic nanolaser devices which are fully plasmonic. In this configuration the air in the MLDA is replaced by another metal film, which generates a fully plasmonic mode and consequently a much greater sub-diffraction limited broadband confinement with no cut-off. Additionally, in order to avoid quenching, a buffer layer is inserted, so technically the previous air layer is substituted by an LM layer, where L is the low index buffer and M is the metal. We have identified in the literature four nanolaser geometries that belong to this category: the nanopillar, the nanopatch, the metal–dielectric cavity, and the coaxial nano-lasers, the latter showing the elusive thresholdless lasing characteristic. Furthermore, the double metal structure makes electrical pumping possible as negative and positive doped junctions may be attached to each metal surface to promote electrical injection.

#### 3.2.1 3D MLDLM nanolaser configuration

The 3D MLDLM configuration nanolaser confines light in all three dimensions to sub-diffraction limited scales and includes the 3D nanopillar laser and the nanopatch laser described more in details in the next section.

##### 3.2.1.1 Nanopillar laser

The original nanopillar laser was demonstrated by Hill et al. [54]; an InGaAs semiconductor was embedded in a pillar between a negative (*n*) doped and positive (*p*) doped semiconductor junctions, and the entire structure coated in gold. The height and radius of the gain medium is 300 nm and 210 nm, respectively, providing light confinement at the nanoscale (according to our definition) in all three dimensions. When pumped, the metal-dielectric-metal interface in the transverse direction along the length of the pillar produces gap plasmon modes which were shown to cause emission in the near infrared of *λ* ∼ 1400 nm. This means that near to subwavelength confinement was achieved in all three dimensions. A diameter increase was found to cause a drastic red shift in the emission wavelength. This work provided the smallest electrically driven nanolaser at the time with a low threshold and operating close to the diffraction limit. The successful electrical pumping of this nanopillar laser lends itself to the MLDLM configuration and the low current required to induce lasing gives rise to possible optoelectronic integration and applications. However, although subwavelength confinement was achieved in the gain medium the overall height of the structure is on the order of a micron. Dense arrays of these structures may however lead to large scale macroscopic output which could compete with other bulk lasers, and this should be further explored. The first nanopillar laser would go on to be the only 3D confined nanopillar example to date.

##### 3.2.1.2 Nanopatch laser

**Figure 16:**
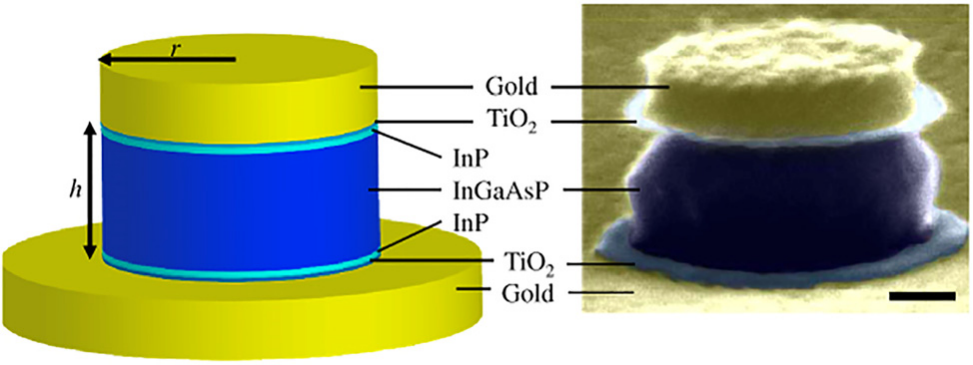
Nanopatch laser structure showing theoretical configuration and SEM image of demonstrated nanopatch [56]. Reproduced with permission. [Optica Publishing Group].

The nanopatch laser is known as an antenna-like nanolaser due to the easy ability to manipulate the output emission radiation. The nanopatch laser was first proposed by Manolatou and Rana [55] in which a gain medium is sandwiched between two metal plates with *n* and *p* semiconductor junctions and insulating spacers separating the junctions from the metal (schematically shown in Figure 16). This structure allows for electrical injection through the metal plates with the *n* and *p* semiconductors encouraging flow through the structure. Lasing occurs by the formation of plasmonic modes between the metal-dielectric interfaces providing excitation of the gain medium and strong confinement. These structures included various gain medium geometries like cylindrical, square, and hexagonal.

Only the cylindrical [56] and square [57] nanopatches have been demonstrated to date with gain medium thicknesses and radii between 230–350 nm and 200–350 nm, respectively. While the high end of these dimensions lies outside of the definition provided in Section 2, both demonstrated nanopatches emit in the near-infrared and are thus are confining light in the deep sub-wavelength regime and as such have been included in this review. One important finding from the initial demonstration by Yu et al. [56] is that altering the geometry of the nanopatch can tune the emission wavelength; the authors reported that by increasing the nanopatch diameter from 200 nm to 223 nm a blueshift emission from *λ* ∼ 1425 nm to *λ* ∼ 1300 nm was measured.

Although nanopatch lasers appear large when comparing them to the previously examined nanolasers, their confinement of near infrared light is drastic, especially when comparing their size to other near infrared emitters such as the nanopillar laser. This provides nanopatch lasers with a high degree of utility for near infrared applications.

#### 3.2.2 2D MLDLM nanolaser configurations

The 2D MLDLM nanolaser configuration confines light in two dimensions within the laser structures leading to larger sizes than those presented in Section 3.3.1. The 2D nanolasers include the coaxial nanolaser, the 2D nanopillar lasers, and the metal-dielectric cavity nanolasers.

##### 3.2.2.1 The coaxial nanolaser (a thresholdless laser)

**Figure 17:**
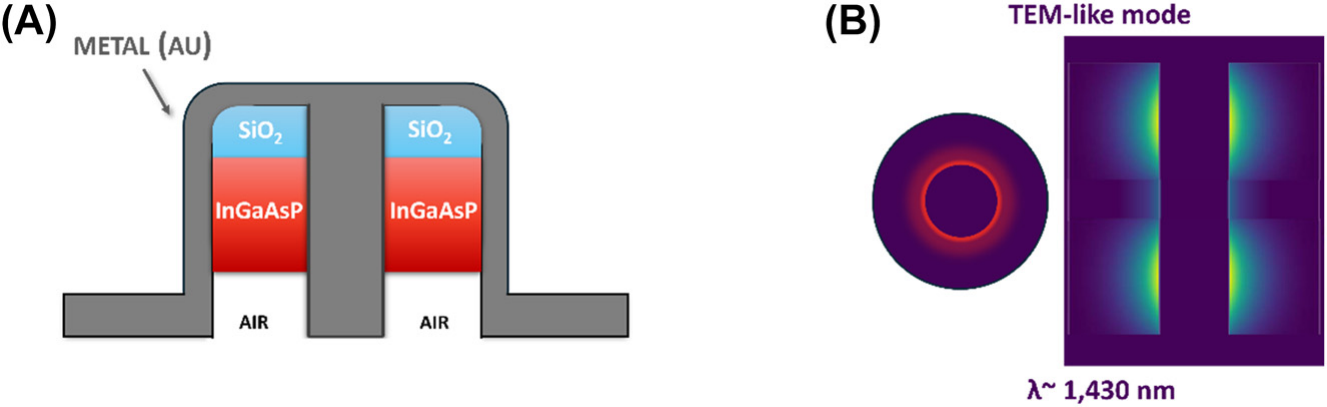
Coaxial nanolaser example. (A) Schematic of the coaxial nanolaser design; (B) approximate representation of the overlap of the plasmonic cavity mode with the gain curve, and cross-sections of the electric field intensity for each mode.

The coaxial nanolaser was first proposed and demonstrated by Khajavikhan et al. [58] and has structure similar to a coaxial cable. At the core of the structure sits a metallic rod (Au), surrounded by the gain medium (InGaAsP), wrapped around the rod which acts like a waveguide. The gain medium is topped by a low index insulator ring to stop plasmonic interactions at the top of the structure and the entire structure is coated with metal leaving an opening at the bottom of the structure for optical pumping (Figure 17A). The gain medium has a thickness and inner core radius of 100 nm each and the height of the gain media is 210 nm. The team optically pumped this nanolaser through the bottom opening at 4.5 K. They found that this nanolaser has no lasing threshold, the highly elusive thresholdless nanolaser had been found. This is due to the nanolaser supporting solely gap plasmon modes between the metal–semiconductor–metal interface transverse to the pumping direction of the laser. Stated in another way, the plasmonic mode is the only mode that overlaps with the gain curve of the active medium, so all pumping acts to create spontaneous emission in the lasing mode (*β* ≥ 0.99), this is approximately illustrated in Figure 17B. As can be seen from the electric field intensity profile in the figure, the lasing TEM-like mode is tightly confined to the inner metal dielectric interface and has an effective mode volume of $0.304{\left(\lambda /2n\right)}^{3}$ .

This coaxial type nanolaser is the only thresholdless nanolaser that has been demonstrated thus far, however operation at room temperature and electrical injection have yet to be demonstrated inhibiting its viability in a range of applications. Furthermore, there is some debate about its truly thresholdless operations since the high *β* factor causes a characteristically lasing-like narrow linewidth [59], since it is more difficult to define a threshold when *β* is large. As reported by Ning [59], in order to correctly evaluate the laser threshold the measurement of the second-order correlation is essential and accurate when *β* approaches 1; in this case for very high pumping the laser appears to be thresholdless, which however is different from a threshold-zero system. Nevertheless, the MLDLM structure may permit these nanolasers to be electrically pumped and if the extremely low threshold can be maintained at higher temperature these devices could prove the ultimate on chip optical component that could be integrated into dense optical systems.

##### 3.2.2.2 2D nanopillar lasers

**Figure 18:**
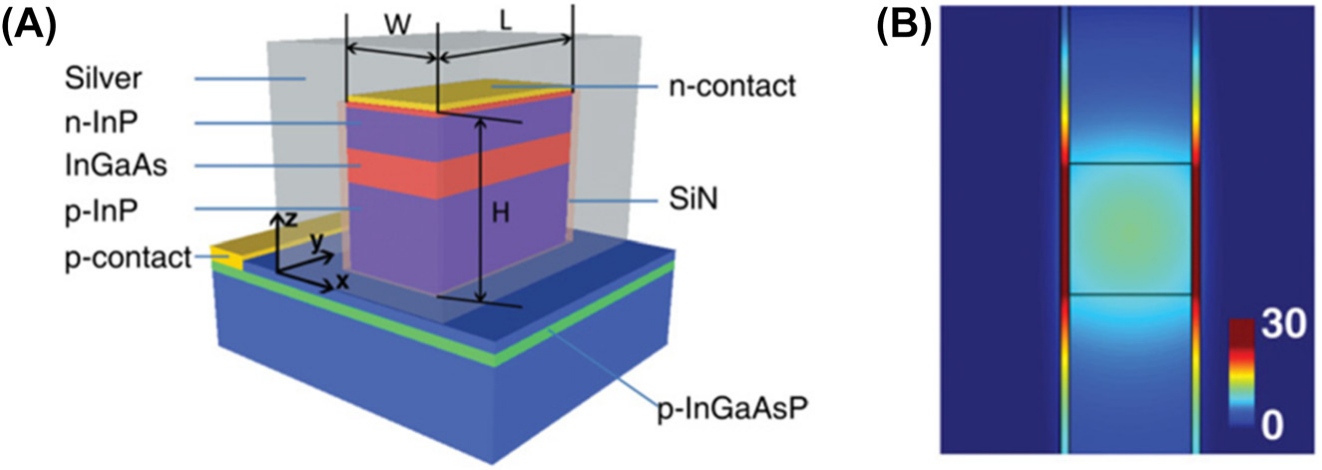
2D nanopillar laser example. (A) 2D nanopillar laser schematic [60]. Reproduced with permission. [American Physical Society]; (B) electric field intensity plot of the transverse cross-section of the gain medium [60]. Reproduced with permission. [American Physical Society].

The 2D nanopillar works in a similar manner to the 3D nanopillar laser. However, these nanolasers support both photonic and plasmonic modes within their cavities. The general construction of these devices involves placing a gain medium inside a vertical pillar sandwitched between *n* and *p* semiconductor junctions; a metal covers the pillar so that it is in contact with the gain medium and insulators that are placed between the junctions and the metal; this is schematically shown in Figure 18A. The junctions allow for electrical injection into the structure which excites the gain medium where both optical and plasmonic modes form. The optical modes propagate along with the gain medium within a Fabry Perot type cavity where the length is on the order of a few wavelengths long, and the plasmonic modes is formed between the metal–dielectric–metal, in the transverse direction, forming gap plasmon modes within the cavity and consequently confining light to sub-wavelength scales (Figure 18B).

Multiple demonstrations of these structures have been performed [60], [61], [62] all presented with emission in the near infrared (*λ* = 1300–1600) nm with subwavelength confinement between 0.42*λ* ^3^ and 0.67*λ* ^3^. Simulations on these devices have also shown that these metal clad nanopillar lasers show promise for direct integration on silicon substrates [63] which would have a wide-reaching implications for on chip integration of these subwavelength infrared emitting light sources.

##### 3.2.2.3 Metallo–dielectric cavity laser

**Figure 19:**
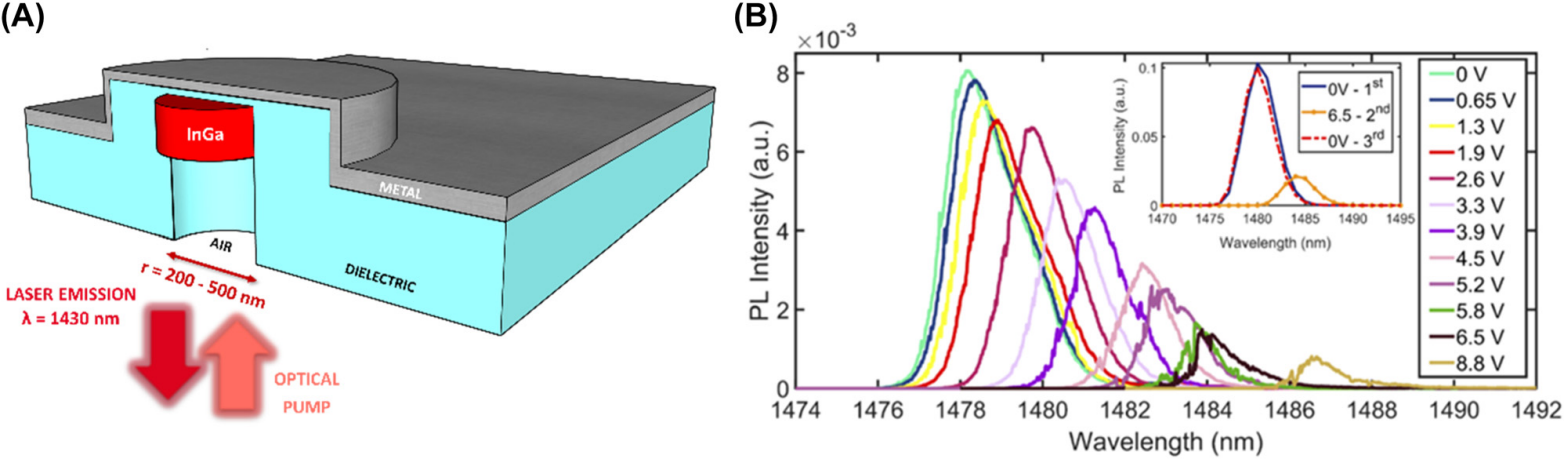
Metallo–dielectric cavity laser example. (A) Schematic of the metal–dielectric cavity laser; (B) real-time wavelength tunability of a metal–dielectric cavity laser [66]. Reproduced with permission. [Optica Publishing Group].

This type of nanolaser is composed of a gain medium which sits in a metal cavity with an open base. An insulator separates the gain medium, this is schematically shown in Figure 19A. The cavity of the nanolaser supports both photonic and plasmonic modes, with the plasmonic modes localized about the metal insulator interface at the top and bottom edges. This structure was first demonstrated to lase using a InGa gain medium under pulsed operation emitting in the near infrared regime at room temperature, with a high-quality factor and moderate lasing threshold [64]. The first reported geometry exhibited a radius of 160 nm and a height between 200 and 500 nm for the gain medium; the lasing wavelength was measured at *λ* ∼ 1430 nm for each geometry, which implies that light is successfully confined in the deep sub-diffraction limited scale.

This structure was later successfully electrically pumped by the introduction of an aluminum oxide dielectric shield layer between the metal and gain, which improves thermal conductivity [65], and a positive terminal layer of ITO was layered at the bottom of the structure, where the metal acts as the positive terminal while the ITO as the negative [66]. The authors further demonstrated that the intensity and wavelength could be dynamically tuned by applying a DC voltage to the structure with a tuning sensitivity of 1.01 nm/V (Figure 19B). Further, the authors found that applying an AC voltage to the structure resulted in the ability to modulate the speed of the laser with a change of 400 MHz recorded in the experiment. The dynamic response characteristics of this nanolaser opens avenues for applications in data transmission and storage at telecom wavelengths and presents a step close to optoelectronic integration. The out of plane lasing action warrants further investigation on macroscopic responses of metal–dielectric cavity arrays which could compete with bulk lasers, with the dynamic tunability of these lasers provides one step closer to nanolaser arrays competing with macroscopic laser sources.

### 3.3 MHDHA and MHDHM nanolaser configurations

The MHDHA and MHDHM nanolaser configurations represent a new class of nanolaser that was theoretically investigated by Li et al. [13]. The team examined all 249 possible configurations of nanolaser waveguide by considering five materials including metal (M), the gain medium dielectric (D), low-index dielectric with respect to the gain medium (L), high index dielectric with respect to the gain medium (H), and air (A). Two of these configurations are the common nanolaser waveguided structures that preceded this section, namely the MLDA and the MLDLM configurations. According to the authors, by simply changing the low index buffer layer “L” with a high index material “H”, the MHDHA and MHDHM structures would exhibit superior performance compared to the classic configurations, such as a highly reduced lasing threshold a much smaller effective area.

**Figure 20:**
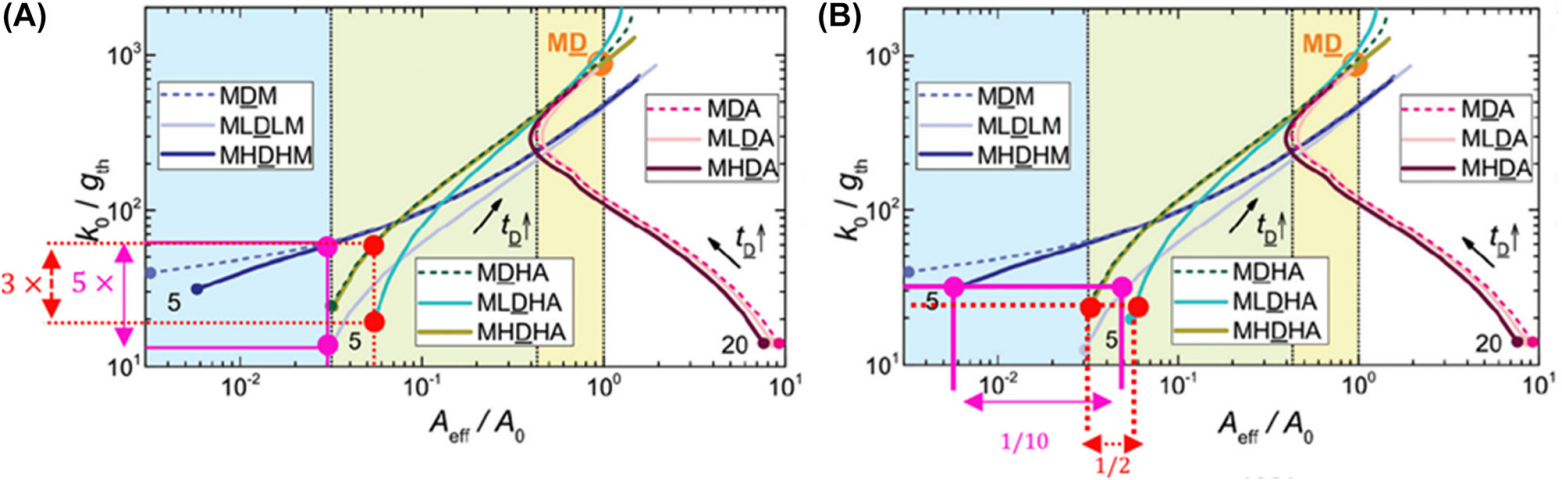
MHDHA and MHDHM nanolaser configurations behaviour. (A) Inverse of the gain threshold versus normalized effective area for configurations of interest for wavelength *λ* = 1550 nm with indicators for increased confinement on structures of interest [13]; (B) same diagram only showing decreased effective area requirement for structures of interest [13]. Reproduced with permission. [© Royal Society of Chemistry].

A high-index material has never been the preferred choice since its optical losses are higher than a low-index counterpart. However, the high index material pushes the propagating mode into the active medium, where it is needed the most, rather than into the low index buffer as in all the previous configurations, hence increasing the achievable gain, which, even considering the additional losses, augments the overall nanolaser efficiency. Figure 20 shows the ratio *k*_0_/*g*_th_ (i.e. the electric energy in the gain medium and that in the lossy metal) where *k*_0_ is the wavevector amplitude in the dielectric and *g*_th_ is the threshold gain versus the normalized effective area *A*_eff_/*A*_0_ where *A*_eff_ is the effective area of the mode and *A*_0_ is the diffraction limited area; the simulations are performed at the wavelength of *λ* = 1550 nm. The results show the MHDHA and MHDHM configurations are superior to their counterparts MLDHA and MLDLM.

Referring to Figure 20A when fixing the effective area (pink vertical line and dots) of the MHDHM (dark blue curve), and the respective MLDLM (grey curve), the *k*_0_/*g*_th_ ratio of the MHDHM configuration is five times higher than the standard MLDLM configuration.

Referring to Figure 20B when fixing the *k*_0_/*g*_th_ ratio (pink horizontal line and dots) of the MHDHM (dark blue curve), and the MLDLM (grey curve), the effective area of the MHDHM structure is 1/10 that of the MLDLM configuration.

Referring to Figure 20A when fixing the effective area (red vertical line and dots) of the MHDHA (khaki curve), and the MLDHA (teal curve), the *k*_0_/*g*_th_ ratio of the MHDHA configuration is three times higher than the standard MHDHA configuration.

Referring to Figure 20B when fixing the *k*_0_/*g*_th_ ratio (red horizontal line and dots) of the MHDHA (khaki curve), and the MLDLM (teal curve), the effective area of the MHDHA structure is 1/2 that of the MLDHA configuration.

**Figure 21:**
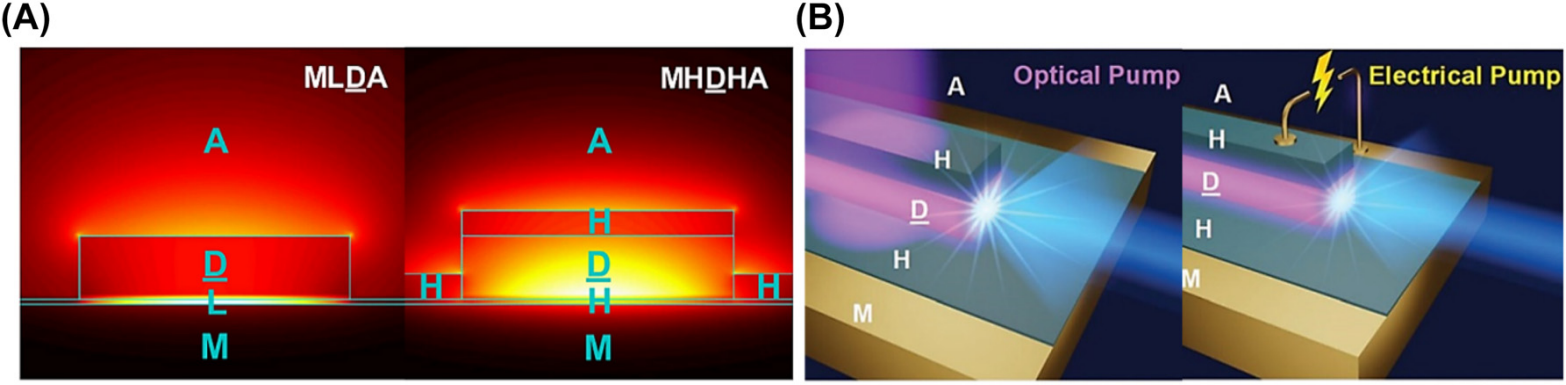
MHDHA and MHDHM nanolaser configurations 2D behaviour. (A) Electric field intensity plot of the MLDA (left) and MHDHA (right) configurations; (B) optical (left) and electrical (right) pumping of the MHDHA structure [13]. Reproduced with permission. [© Royal Society of Chemistry].

In conclusion the MHDHA and MHDHM configurations are superior in *k*_0_/*g*_th_ ratio and in smaller effective areas compared to conventional plasmonic configurations, naturally leading to lower gain thresholds and smaller mode confinements. The MHDHA structure sees a reduction in gain threshold of over 30 % with mode area as small as $0.18\left(\lambda^{2}/2n\right)$ . This new configuration allows far greater light interactions with the gain medium than the conventional low index configurations in which the propagating modes are tightly confined into the low index region (Figure 21A). Furthermore, the introduction of the high index dielectric, instead of the low index dielectric potentially allows electron/hole injection and therefore potentially electrical pumping, as schematically shown in Figure 21B. The MHDHM structure claims an order of magnitude reduction in mode area and a significant threshold reduction, making it far superior structure to the MLDLM structure. Since this is an MDM structure electrical pumping should be possible. The lower threshold and stronger confinement may lead to smaller more efficient devices that could be utilized in many applications and represents an important step towards the goal for low power, small, and electrically pumped devices and as such should be a priority to demonstrate.

### 3.4 Other configurations of interest

Apart from the configurations mentioned above there are other structures that do not fit into any of the configurations, the most important of these include the SPASER, a 3D nanolaser and the stopped-light phenomenon laser, each of these will be briefly described in the next section.

#### 3.4.1 The SPASER

A SPASER (an acronym for surface plasmon amplification by stimulated emission of radiation) uses localized surface plasmons as opposed to SPPs or hybrid plasmonic modes, to amplify light–matter interactions and therefore lasing. They are subwavelength structures in all three dimensions and represent the smallest nanolaser demonstrated to date.

**Figure 22:**
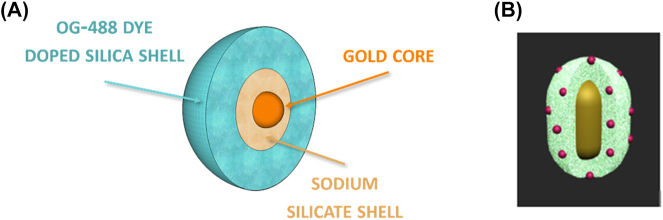
Spherical type SPASER composition; nanorod type nanolaser SPACER [68] [© 2013 American Chemical Society].

SPASERs are purely plasmonic devices that use coherent localized surface plasmon oscillations to induce lasing. The first spasing action was demonstrated in 2003 in a V-shaped groove surrounded by a gain medium. When the gain medium was pumped, resonance surface plasmon modes were detected as highly intense coherent light [9]. It was this first demonstration of spasing action that led to the first nanoparticle SPASER [67]. This SPASER was composed of a gold nano spherical core covered in a low index insulator of radii 13 nm and 14 nm respectively. This structure is then covered in a dye doped gain media as shown in Figure 22A. The insulator acts in the same way as the other configurations examined here, i.e. limiting quenching. When this nanoparticle SPASER was optically pumped, the characteristic narrow laser-like linewidth was observed in the visible at *λ* = 531 nm; this was the smallest nanolaser reported at that time and the first operating at visible wavelengths. This new type of nanolaser produced light that was non-directional, with a relatively high threshold due to high dissipative losses, and a short lifetime which produced an initial impasse on their viability.

Since their first demonstration subsequent experiments have shown that directionality, improved lasing lifetime, wavelength tunability, and a low threshold are all possible in SPASERs. Directionality was achieved by constructing SPASERs with a nonsymmetric shell-core structures achieving an almost unidirectional output [69]. An improved lasing lifetime was achieved by introducing a three-level system as gain medium of the SPASER [70], which also supply a way to engineering the band gap, which may reduce gain thresholds into the future. Wavelength tunability was achieved in a nanorod SPASER, schematically shown in Figure 22B, in which control over the doping of the gain medium dye led to wavelength tunability in the visible between *λ* = 562–627 nm [68]. Large gain thresholds still remain a problem due to large losses in SPASERs, however theoretical low threshold SPASERs have been proposed [71]. SPASERs are an important class of nanolaser due to their small size and range of possible applications.

#### 3.4.2 Stopped-light nanolaser (the cavity free nanolasers)

To conclude our review on the nanolasers types, the most intriguing design is the stopped-light nanolasers also known as cavity free nanolasers. These types of nanolasers have no cavities but light is trapped by closed optical paths within a gain medium which increases the density of optical states inducing stimulated emission and lasing. Two types of these stopped light configurations have been proposed and only one demonstrated.

**Figure 23:**
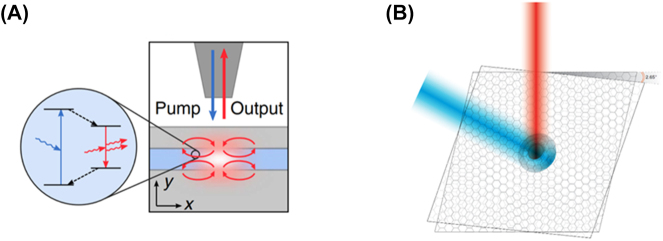
Stopped-light nanolaser examples. (A) The theoretical metal-dielectric-metal stopped light phenomenon laser schematic [72] [Springer Nature, Creative Commons Attribution 4.0 International License]; (B) the demonstrated double graphene stopped light phenomenon laser schematic [73].

The first type of stopped light laser is still currently theoretical and consists of a dielectric that sits between two metal slabs [72], see Figure 23A for a schematic of this design. When the dielectric slab is of a certain thickness the energy flow of the dielectric directly opposes that of the metal, as they have opposite signs in their permittivity, which generates small, closed loop paths that the photons travel causing stimulated emission under pumping. This system is thought to be highly sensitive to surface roughness and has still not been demonstrated. As the light is trapped within the structure directly under the pump in small, closed loops it would be considered a 3D nanolaser.

The second type of stopped light nanolaser is created by placing two superlattice graphene sheets on top of one another at a “magic angle” of 2.65° [73], (Figure 23B). At this angle the group velocity of the electrons moving between the two sheets becomes zero allowing for unique excitation properties including stimulated emission through closed stopped light loops. The confinement is in one dimension making this a 1D nanolaser. The field is too new to know the full characterization of these nanolasers, but possible macroscopic lasing appears promising from both types mentioned here.

## 4 Nanolaser arrays and their properties

Nanolaser arrays are large sets of nanolasers that act together to produce a larger effect, potentially larger than the mere sum of the individual outputs. In this review we are mainly considering arrays constituted by individual nanolasers which act independently if removed from the array, they are stand-alone structures that operate independently from the surrounding. This does not stand for nanolasers engineered specifically to operate as sensors, like those described in Section 5.1, where the individual characteristic of the nanolaser is altered by the presence of a molecule and hence by the environment. This definition differs from lasing based on all-dielectric/plasmonic metasurfaces and especially based on surface lattice resonances and the quasi-bound states in the continuum, since their control and modulation of light is due to their overall engineered permittivity and permeability. These structures have to date reported phenomenal results and advancements. Issues like spatial coherence, losses, lasing threshold, wavelength tunability, and directional emission that challenge waveguide-based nanolasers have been well solved by metasurface-based nanolaser arrays [74], [75], [76].

**Figure 24:**
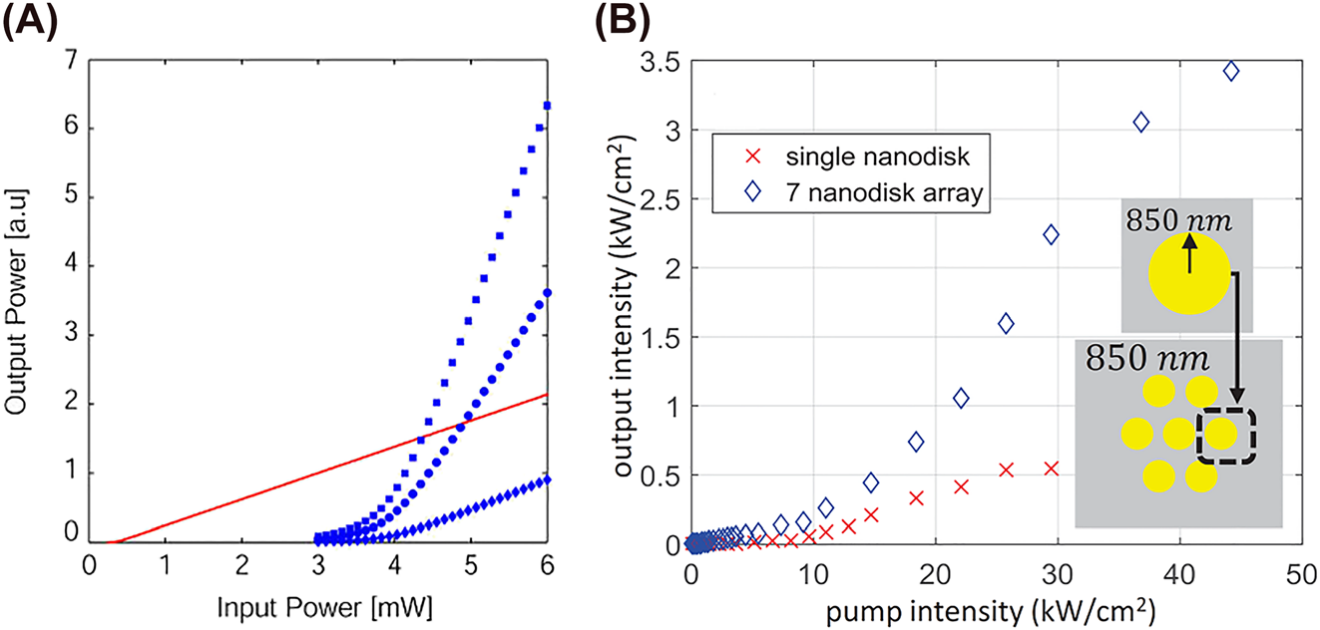
Nanolaser array properties. (A) Analytical comparison of output power versus input power for a single photonic crystal cavity laser (red), and array of 10 photonic cavities (blue-diamond), an array of 40 photonic cavities (blue-circle), an array of 70 photonic cavities (blue-squares) [77]. Reproduced with permission [Optica Publishing Group]; (B) experimental comparison of the input-output curve for a single nanodisk laser (red) compared to a 7 nanodisk array (blue) [78].

The field of nanolaser arrays is currently in its infancy and as a result there are limited demonstrations of these nanolaser systems to date. These laser arrays may be broadly characterized as being (i) uncoupled, or (ii) coupled. Uncoupled nanolaser arrays are groups of nanolasers whose modes do not interact with each other but produce a collective effect based on the collective sum of the individual nanolasers. Coupled nanolasers on the other hand are made of groups of nanolasers whose modes interact with each other in a way that enhances the properties of the nanolasers, these collective modes are known as supermodes. Uncoupled nanolasers are better suited to applications in which mode interaction would be detrimental for operations such as data storage and transmission, in case each unit acts as a data processor. These arrays do not improve in efficiency with increasing the unit number. Coupled nanolasers are better suited for applications in which efficiency becomes important and manipulation of the output field is desired, as each individual unit may improve the efficiency and output of those surrounding it. Both analytical and experimental results have shown that coupled nanolasers can produce non-linear power relationships compared to uncoupled nanolasers, ultimately increasing power output for the same power input. Altug and Vučković [77] analytically demonstrated that arrays of photonic crystal laser cavities outperformed single unit laser cavities for equal power inputs, above a threshold for arrays of 40 and 70 cavities, respectively. Figure 24A shows that the single laser unit (red) exhibits a non-linear response between input and output powers where the greater the number of units in the array the greater the benefit. Hayenga et al. [78] experimentally demonstrated this non-linear coupling relationship in nanolaser arrays for nanodisks. The team compared a single nanodisk laser to an array of 7 nanodisk nanolasers and found that for the same pump powers the array outperformed the single laser at an increasing nonlinear rate, (Figure 24B).

In our opinion, this confirmation of the non-linear relationship in coupled arrays presents a wide range of opportunities for low power/threshold applications and may lead to coupled nanolaser arrays outperforming bulk nanolasers which in turn may lead to the next generation of macroscopic laser devices useful in the span of applications that it could have compared to the present technologies that might require miniaturized yet high power laser devices. This section will provide a brief overview of the current state of nanolaser array research. Our definition of nanolaser will be used throughout this section and will be determined by the individual lasing unit for each of these arrays.

### 4.1 Nanowire and nanoslab arrays

Nanowire (2D type nanolaser) and nanoslab (1D type nanolaser) arrays have become active areas of research in recent years with two main array types, the single nanowire/slab on a grating array, and the bus nanowire array. Both array types are uncoupled in that the lasing from each single unit does not affect the lasing output of other units in the array. Within this nanolaser array type the IDA and MLDA configurations are taking advantage of both photonic and hybrid photonic lasing.

**Figure 25:**
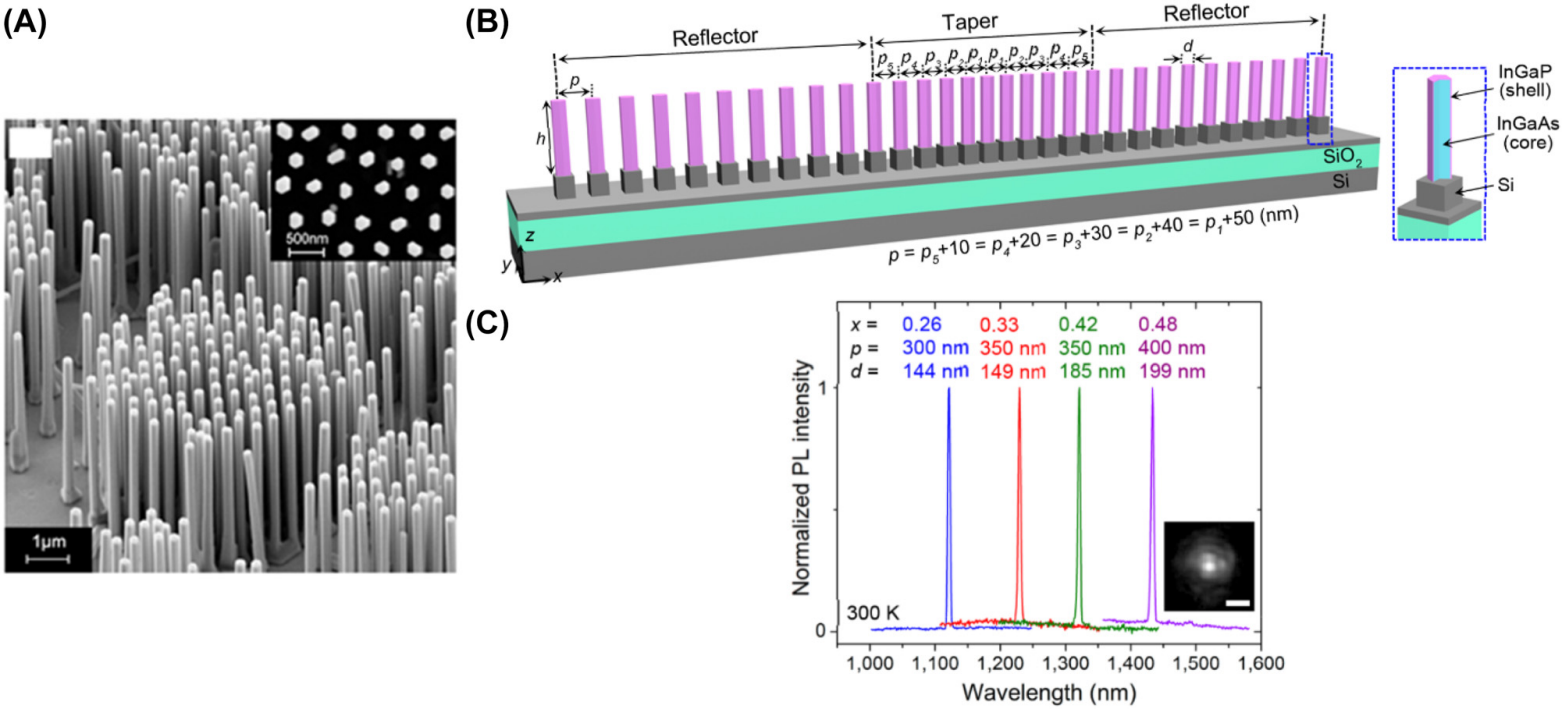
Nanowire array nanolaser example. (A) ZnO nanowire arrays grown on Al2O3 [79]; (B) schematic illustration of an InGaAs/InGaP core/shell nanowire array on an SOI platform [80] reproduced with permission [ACS Publications]; (C) lasing spectra of nanowire array cavities on each sample. Inset: emission pattern above lasing threshold showing an interference fringe pattern. Scale bar, 5 μm [80]. Reproduced with permission [ACS Publications].

The single unit nanowire array consists of uncoupled nanowires that are grown either vertically or horizontally on a substrate. Since the emission is out of the end facets of the nanowires the vertical array can produce out of plane lasing. This was demonstrated by Zhou et al. [79] in which ZnO nanorods were gown vertically on a substrate using a controlled vapor method, this array is presented in Figure 25A. They found that these nanolasers produced a large macroscopic response, emitting in the UV and producing out of plane emission with possible applications in sterilization. Greater control of nanolaser length will be required to produce monochromatic light.

Recently, a 1D line array of nanowires were constructed [80] and shown to be compatible with growth on silicon, control over the length of the nanowires acted to tune the wavelength of the device. This monolithic device provides a step towards chip integration and data communication, as such further research on the macroscopic response of the nanowire array should be conducted. Another more unique application for single unit nanowire arrays is in creating cryptographic primitives used in encoding. Feng et al. [81] used an evaporation method to create nanowire lasers of random length but fixed diameter. This results in the nanolasers emitting at 660 nm or 720 nm or both. He found that the randomness of the lasing emission could be used as an encoding sequence.

The nanowire/nanoslab on a grating array type places a gain medium over a grating in either the IDA or MLDA configuration depending on the photonic and/or plasmonic nature of the array.

**Figure 26:**
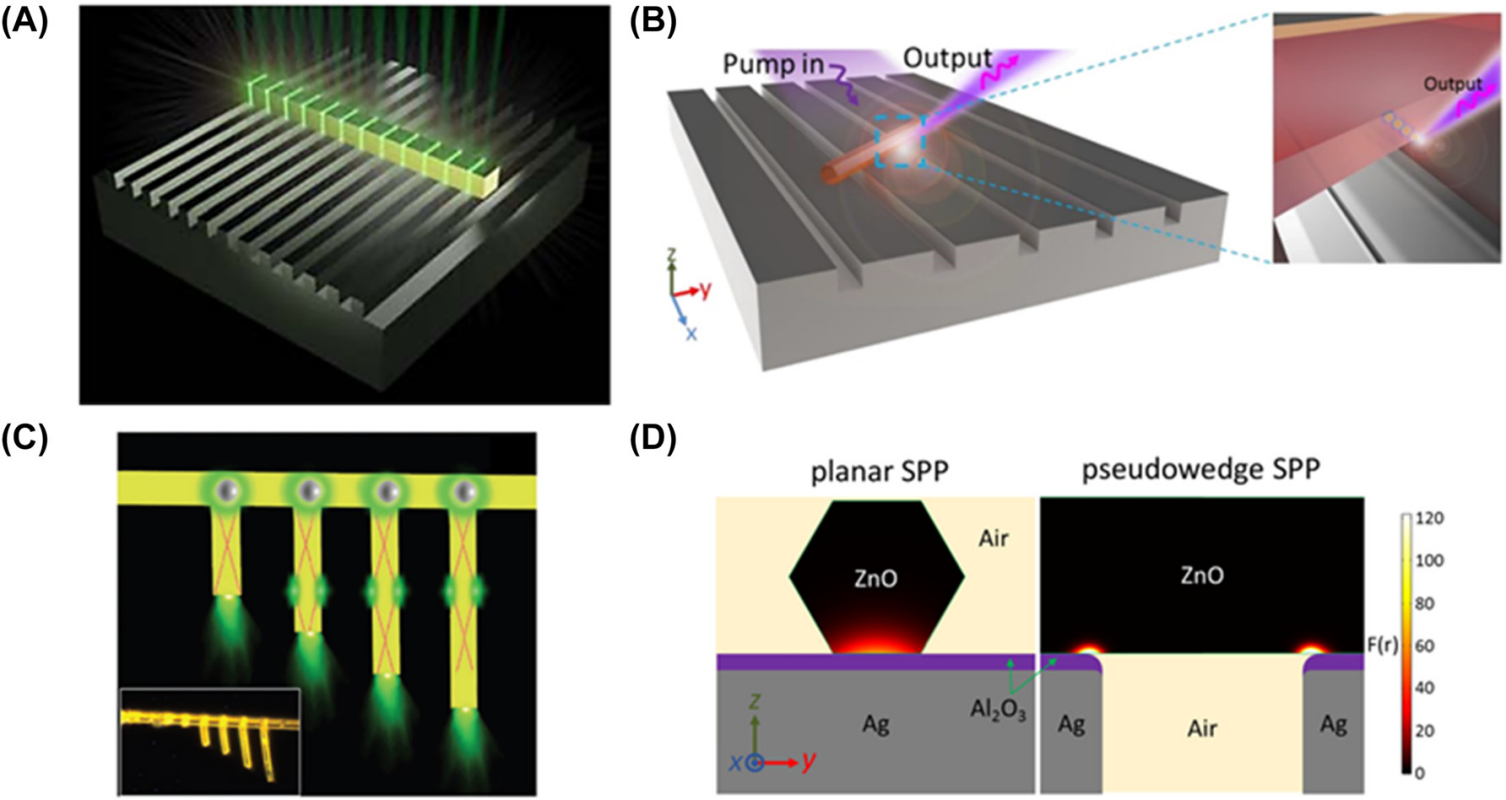
Nanowire array nanolaser examples. (A) Photonic single nanowire array showing lasing [82]. Reproduced with permission [ACS Publications]; (B) plasmonic single nanowire array showing lasing [84]. Reproduced with permission [ACS Publications]; (C) branched nanowire waveguide laser with embedded nanoparticles [85]. Reproduced with permission [© 1999–2023 John Wiley & Sons, Inc.]; (D) comparison of planar and pseudo-wedge electric field confinement suspended regions in this type of nanolaser are responsible for lasing and as such form the array [84]. Reproduced with permission [ACS Publications].

The first array of this type was demonstrated by Wang et al. [82] and consisted of a methylammonium lead bromide nanowire placed over a silicon rectangular groove grating, this is shown in Figure 26A. They found that the suspended region over the grating has a high-quality factor compared to the non-suspended regions and acted like small individual Fabry Perot cavities allowing for lasing. This photonic type nanolaser array produced out of plane lasing, successfully demonstrating that single nanowires can use substrate geometries to generate arrays, which has wide reached implications for out of plane optical interconnects and waveguiding of laser light. Plasmonic versions of this nanolaser array have also been demonstrated using semiconductors separated from a metal by an insulator, first demonstrated with a nanoslab [83]. Chou et al. [84] used a ZnO nanowire place on a silver pseudo wedge type grating and found that high intensity lasing was observed, emitted from the points of contact of the nanowire with the suspended region, as schematically shown in Figure 26B. By comparing the electric field confinement of a regular ZnO nanowire on a planar plasmonic surface, as discussed in Section 3, to the field confinement on the pseudo wedge grating, the team found that plasmonic action at the points of contact were enhanced with exceedingly small mode volumes, as shown in Figure 26D. This feature of plasmonic gratings could be utilized for optical interconnects and if placed in large arrays for far field applications.

The final type of nanowire array is a hybridized structure using a branched nanowire gain medium with embedded metallic-dielectric nanoparticles, first introduced by Hao et al. [85] as schematically shown in Figure 26C. The waveguide acts as a standard Fabry–Perot cavity, where the nanoparticles enhance light matter interaction and scatter light into the branches. They showed lasing out of multiple branches which could be used in light guiding.

### 4.2 Nanoparticle arrays

Nanoparticle arrays incorporate large numbers of nanoparticles, usually in different structures to stand alone nanoparticle lasers, to produce large out of plane effects. These arrays differ from the nanowire/nanoslab arrays of Section 4.1 as they couple, enhancing or degrading the array output. A variety of nanoparticles have been demonstrated in arrays including SPASERs, dimers, and nanorods.

SPASER arrays have been of interest in modern array research as they easily couple together through dipole-dipole interactions causing synchronized outputs. This synchronization has been theoretically shown to enhance the field in what is known as superradiance of SPASERs in 1D chains [86], and 2D planes [87].

**Figure 27:**
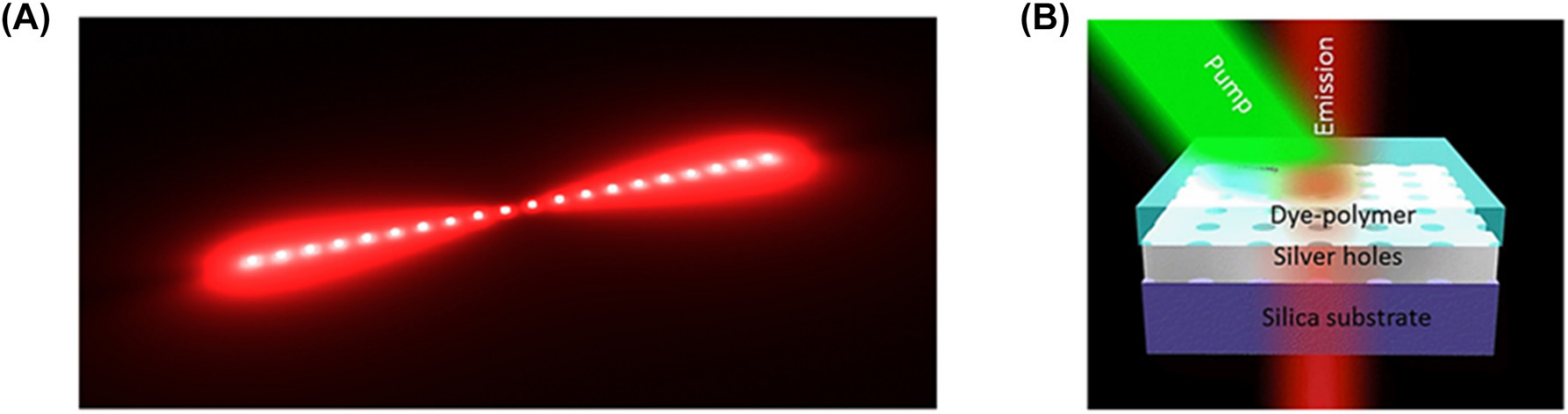
1D and 2D SPASER array examples. (A) 1D coupled SPASER array [88]. Reproduced with permission [ACS Publications]; (B) 2D coupled SPASER array [89]. Reproduced with permission [© 1999–2023 John Wiley & Sons, Inc.].

The one-dimensional chain of SPASERs was demonstrated by Rekola et al. [88] in which small silver cores were gown on a substrate and covered with a laser dye gain medium, as shown in Figure 27A. When pumped lasing was observed in the red with a reduced threshold than the individual units separately demonstrating the coupling of SPASERs along the chain. Although each SPASER is a 3D nanolaser the chain array represents a 2D equivalent nanolaser.

The two dimensional SPASER array was demonstrated by Meng et al. [89] and consisted of a metal film with nanoholes built in to the structure, the SPASERs sit within the nanoholes and the entire structure is covered with a dye gain medium, as shown in Figure 27B. The authors showed that when inserted into this configuration SPASERs act with a single enhanced mode, emit in the visible, and this light is directed out of the plane. This configuration shows superradiance of a 2D arrays of SPASERs, which act like a 1D nanolaser and may have applications such as macroscopic far field lasing.

**Figure 28:**
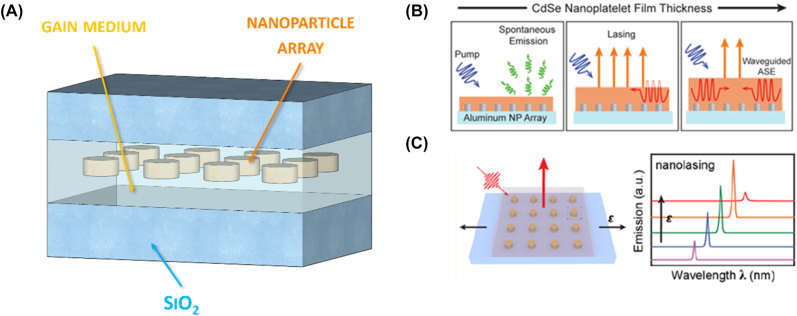
Metallic nanoparticle array examples. (A) Metallic nanoparticle array in an IMDA configuration; (B) metallic nanoparticle array in an MDA configuration [92]. Reproduced with permission. [ACS Publications]; (C) wavelength tunable metallic nanoparticle array [91]. Reproduced with permission. [ACS Publications].

Another type of nanoparticle array is composed of metallic nanoparticles grown on an isolator or metal substrate with a covering gain medium layer. These types of arrays thus fit a MDA (metal–dielectric–air) or IMDA (isolator–metal–dielectric–air) configuration, Figure 28A and B present an IMDA and MDA array structure, respectively.

Zhou et al. [90] demonstrated the first metallic nanoparticle array in which silver or gold nanoparticles were grown on a glass substrate and covered with an organic gain medium which itself was covered in glass, as shown in Figure 28A. The gain medium acts to transfer energy to plasmonic modes within the metallic nanoparticles which in turn cause lasing. They found that the emitted light from the nanoparticle array was highly directional propagating out of the plane with a less than 1.5° divergence and 1.3 nm linewidth. Furthermore, a wavelength tunable strain gauge set up has recently been theoretically proposed [91], as shown in Figure 28C. This initial demonstration along with the possibility for wavelength tunability showed metallic nanoparticle arrays have great potential for far field macroscopic applications.

The MDA plasmonic configuration of this set up was first demonstrated by Watkins et al. [92] in which CdSe nanopellets were grown on an aluminum planar surface and covered in a dye gain medium, as shown in Figure 28B. Depending on the thickness of the gain medium the array would support either surface plasmon modes within the cavity or hybrid plasmonic photonic modes within the cavity. Light is emitted out of the plane with a greater intensity approaching 150 nm in thickness and no lasing below that thickness. Other variations of this set up have also been proposed [93] with a variety of far field applications.

**Figure 29:**
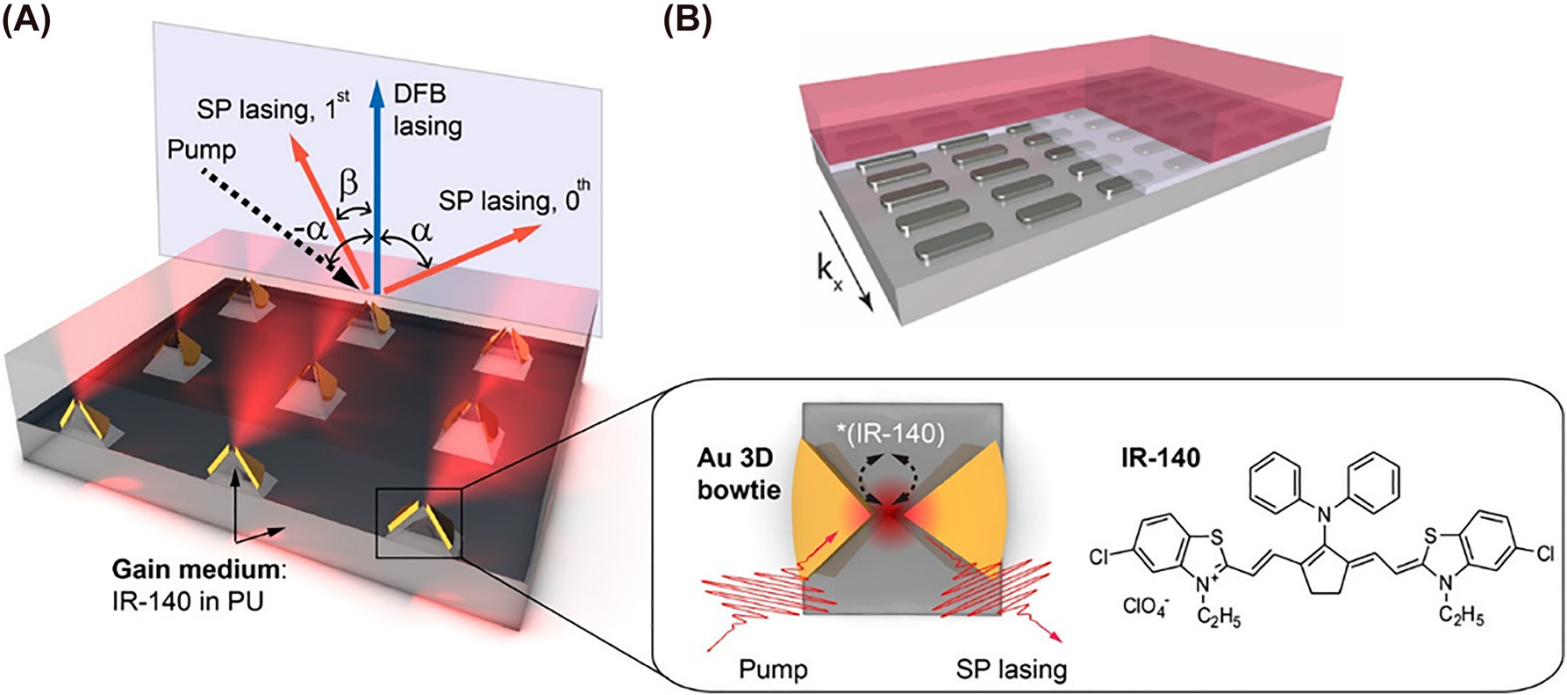
Bowtie array nanolaser examples. (A) Bowtie array with insert showing the bowtie nanoparticle structure [94]. Reproduced with permission. [ACS Publications]; (B) plasmon-exciton-polariton nanolaser array [95]. Reproduced with permission. [Optica Publishing Group].

Bowtie nanolasers are a type of nanostructure that are known as dimers. They consist of two bows separated by a gap (in the shape of a bowtie) that support gap plasmons with a high intensity within the gap. These types of nanolaser have been demonstrated in a coupled array structure [94]. The bowtie nanoparticles are grown on a substrate in a periodic structure with a dye gain medium covering the structure; this is shown in Figure 29A with a schematic of the bowtie structure in the insert. Upon pumping it was found that surface plasmonic modes caused lasing emission in two oblique directions and band edge lasing was observed transverse to the plane with ultrafast response times. The oblique and transverse lasing directions coupled with the ultrafast dynamics could provide useful for compact biosensors and light coupling applications in the future.

Another nanoparticle array is the plasmon–exciton–polariton (PEP) nanoparticle array and was experimented by Ramezani et al. [95]. This array consisted of silver nanoparticles that grown on a polymer substrate covered in a dye doped gain medium. This nanolaser, under optical pumping, demonstrated out of plane lasing with a small threshold. More interestingly, they found that when light–matter interactions increase, a decrease in threshold was recorded, which is counterintuitive as higher losses are expected with greater light matter interactions. They believe that the coupling of modes within this system caused this counterintuitive interaction. Further investigation on arrays like this may increase our understanding coupling in arrays. This is chemotactically shown in Figure 29B.

An experiment carried by Hakala, T. K., et al. [96] reports of the physical conditions for the manifestation of macroscopical quantum coherence, by carrying some comparative measurement on the selected arrays of plasmonic nanoparticles. They demonstrated a Bose–Einstein condensate (BEC) of surface plasmon polariton in lattice modes, remarkably they extract a corresponding band structure, depending on the geometry of the array, and they tailor and observe a cross-over from a BEC to lasing mode. This aligns with what has been mentioned earlier comparing that lasing was realized in similar systems of plasmonic lattices in the weak coupling [90] and the strong coupling [95] regimes. To compare surface lattice resonance lasing with BEC, they realized a setup which accesses the sub-picosecond dynamics of thermalization and condensate formation that allows broader insights of how to engineer the mechanics of those systems.

Other nanoparticle arrays have been theoretically proposed such in which a planar metamaterial gain medium with small arrays of small nanowire rings cause low divergence out of plane lasing [97].

### 4.3 Nanoantenna arrays

**Figure 30:**
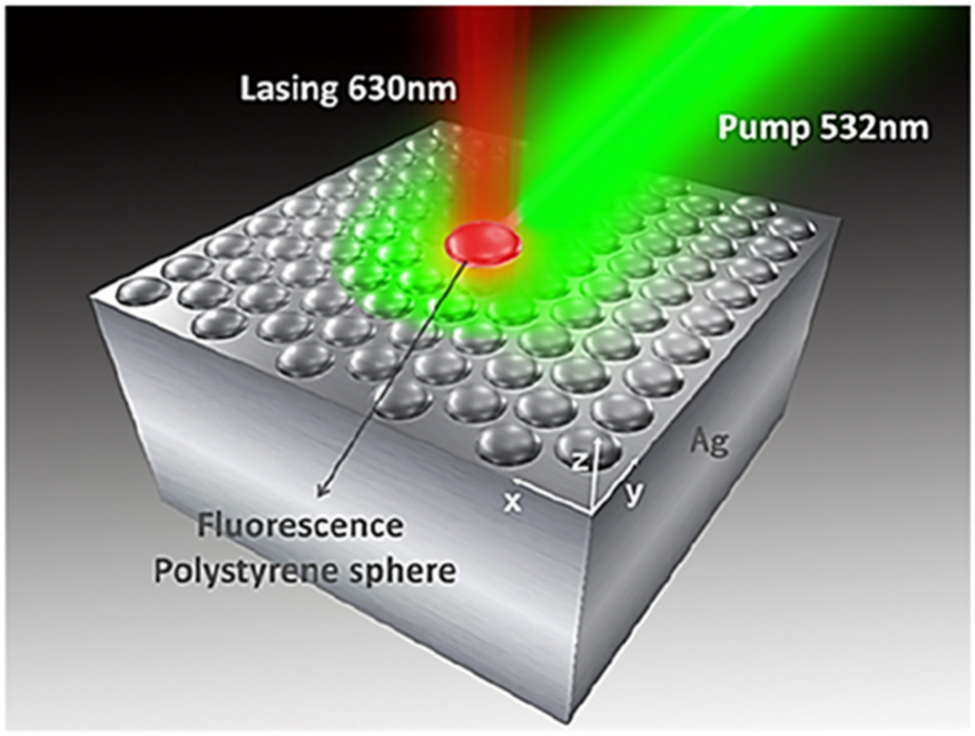
Plasmonic nanoantennae array [98]. Reproduced with permission [ACS Publications].

The plasmonic nanoantenna array was first demonstrated by Zhang et al. [98] in which polystyrene sphere were used to cover metallic nanoparticles i.e. (the nanoantennae) (Figure 30). When pumped, the spherical shape of the polystyrene sphere would guide light within the cavity. Plasmonic modes are formed between the edges of the spheres and the nanoantennae, tightly confining light; this was found to reduce plasmonic losses that usually plague metallic nanoparticles. Their results showed a twenty-fold decrease in threshold compared to similar nanocavity lasing arrays, mode volumes as small as $0.22{\left(\lambda /2n\right)}^{3}$ and transverse lasing emission. This result shows great promise for the use of these nanolasers in applications such as sensing and data communication.

The final nanolaser array was demonstrated Ha et al. [99] in which nanoantenna arrays were grown on a quartz substrate and with a hardened solution of hydrogen silsesquioxane. When optically pumped these nanolasers showed highly directional diffraction-based lasing which could be manipulated by changing the antennae geometry.

The wide range of properties that have been observed in nanowire, nanoparticle, and nanoantennae arrays provide promise for a wide range of applications from low threshold macroscopic responses, beam manipulation, and dense optical integration.

## 5 Nanolasers and their applications

One of the reasons of interest in nanolasers is the wide reach of possible applications that a low threshold, tunable, electrically pumped, and very compact laser could allow. This section will cover some of the most important nanolaser applications demonstrations and some theoretical possibilities that these nanolasers may soon encompass.

### 5.1 Nanolaser sensors

Currently, plasmonic sensors are the branch that has seen the majority of commercial applications to date. These sensors are used for detection of molecules in compact devices by using surface enhanced Raman scattering (SERS); surface plasmon resonance to detect molecules in very small concentrations in biological and chemical sensing [100], [101] as well as passive pregnancy and antigen (e.g. SARS-CoV-2) tests. However, SERS sensors still require lasers and spectrometers to function, which are still much larger than the sensor itself, making the potential “lab-on-a-chip”, an actual “chip-in-the-lab”. The great benefit of using nanolasers for sensing applications is that their tight plasmonic mode confinement and temporal coherence leads to be very sensitive to small changes in the environment which reflect into measurable radiative output variations [102], [103], on top of being integrable on the same chip.

**Figure 31:**
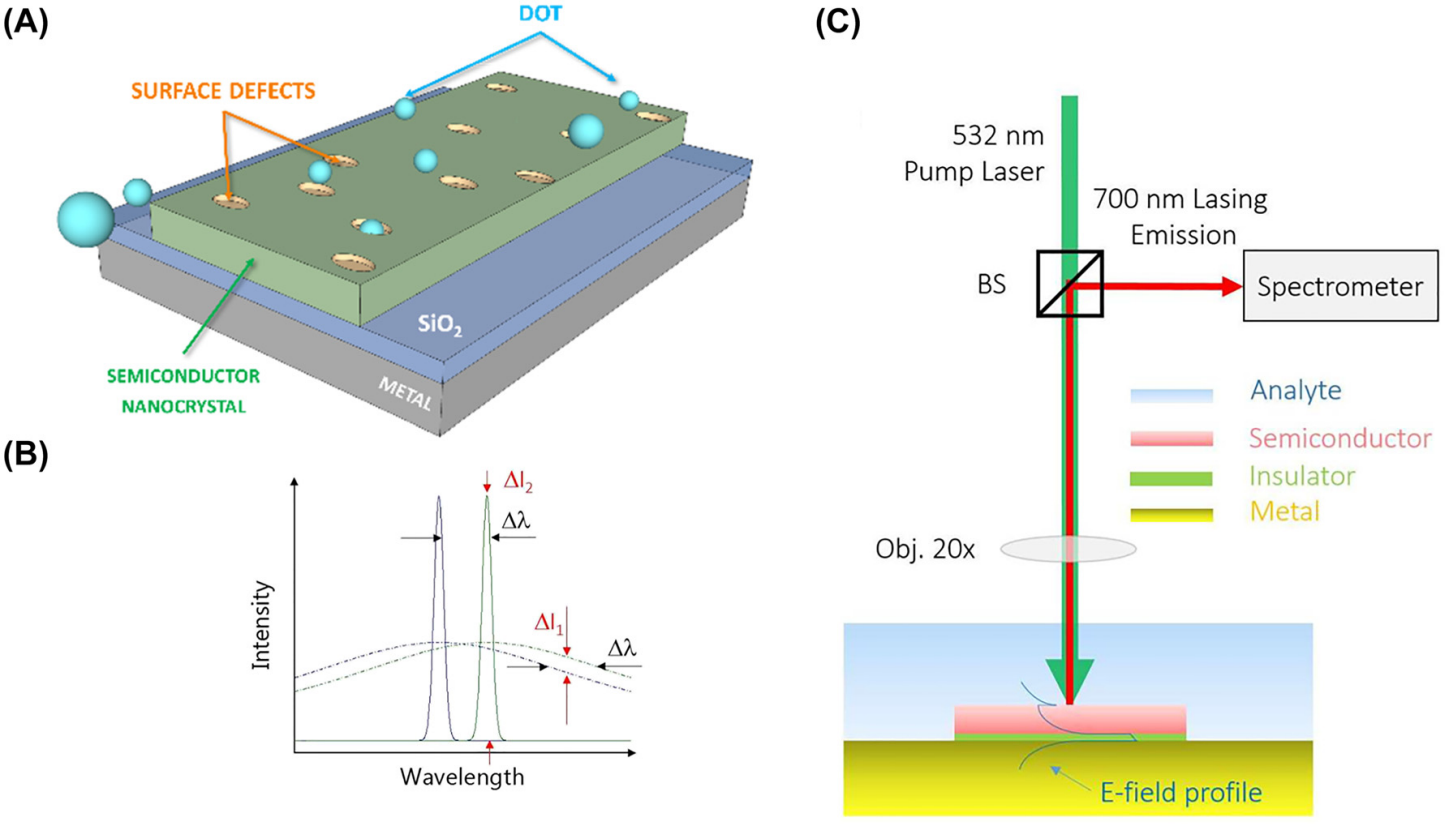
Nanolaser sensor examples. (A) Gas detection nanolaser schematic; (B) lasing enhanced surface plasmon resonance versus surface plasmon resonance in refractive index sensor [105]; (C) schematic of refractive index nanolaser sensor [105]. [DeGruyter, Creative Commons Attribution-NonCommercial-NoDerivatives 3.0 License].

Detection of single molecules is highly important in a range of fields and could significantly improve safety in food quality, in gas sensing, in medicine, and in chemistry. A nano-slab plasmonic nanolaser (a 1D type nanolaser) was used to detect explosive molecules in ultralow concentration as low as 1 part per billion [104], schematically shown in Figure 31A. The system has natural defects on the top surface of the nano-slab, which can be filled by molecules when exposed to certain gas, hence causing characteristic changes in the nanolaser output intensity. They were able to detect the difference between three types of explosive gas molecules both in nitrogen filled and air-filled chambers. This type of nanolaser sensor could eventually serve as sensitive gas detector in a wide variety setting. A large amount of work still needs to be conducted to determine if these nanolaser units can be used in environments at atmospheric pressure.

The second type of sensor we have chosen to highlight is a refractive index sensor and clearly demonstrates the much greater resolution of lasing enhanced surface plasmon resonance (LESPR) over conventional enhanced surface plasmon resonance (SPR). The first demonstration of a laser based refractive index sensor consisted of a MLDA plasmonic configuration of a CdSe nano-slab (1D nanolaser) which is optically pumped [105], this is shown schematically in Figure 31C. When under excitation it was observed that the presence of an analyte solution with a higher refractive index than air caused the emission peak to redshift, hence providing a reliable way to categorize materials refractive indices.

The narrow linewidth of the plasmonic laser emission, when compared to the much broader surface plasmon resonance, allows for much greater accuracy in the detection of refractive index changes. This characteristic is shown in Figure 31B where a shift in lasing emission (high intensity narrow peaks) is compared to the shift in SPR emission (low intensity broad peaks). In this first demonstration, a 0.3 nm redshift in wavelength was measured when comparing the refractive index of ethanol (1.3588) with propyl alcohol (1.3801); this sensor claimed to be 400 times more sensitive than the most advanced surface plasmon resonance sensors. These devices, however, were shown to be easily damaged by water, hence reducing their biological applicability. This issue was later mitigated by introducing an aluminum oxide coating over the gain medium of the device increasing aqueous compatibility from ∼20 % to ∼70 % showing stable lasing after 3600 s [106]. The improved suitability of this type of nanolaser in biocompatible environments makes it highly attractive.

Since the original demonstration a variety of refractive index sensors and sensor arrays have been demonstrated using nanowires on gratings [107] and plasmonic trench nanocavity lasers [108].

**Figure 32:**
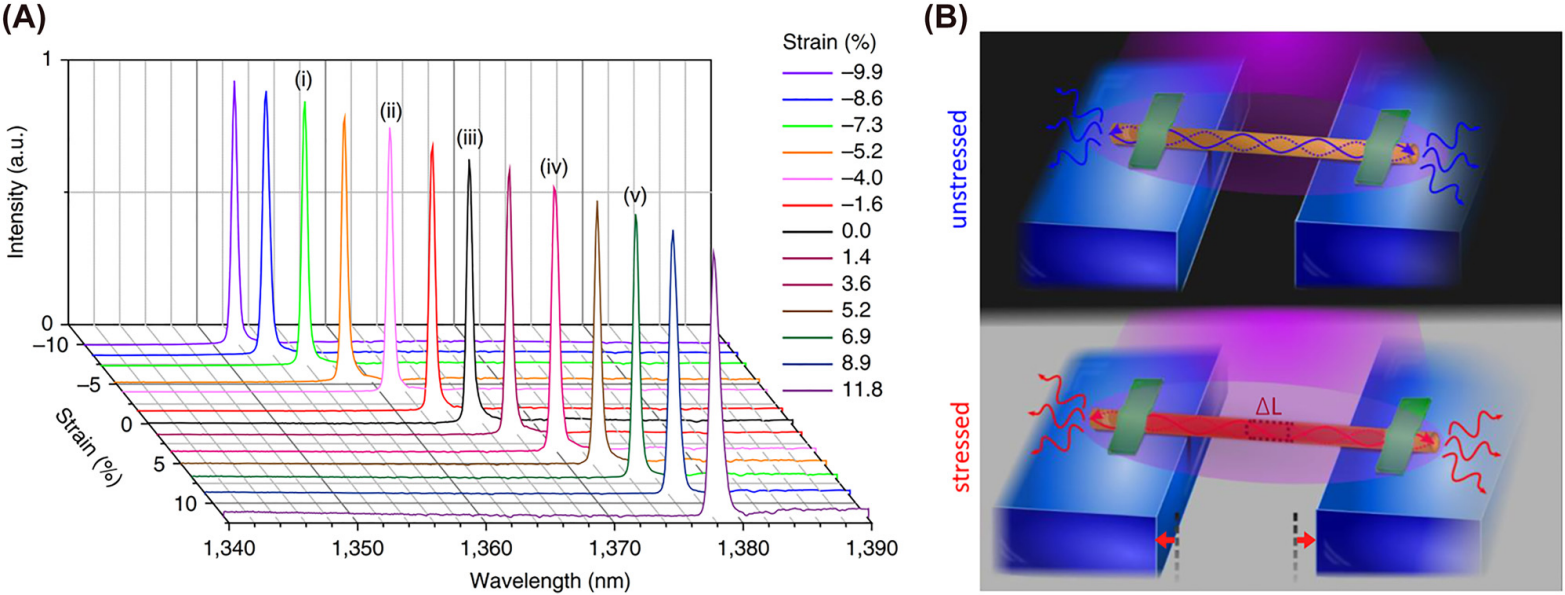
Strain gauge nanowire sensor. (A) Strain gauge response of a photonic crystal array strain gauge [109] [Springer Nature, Creative Commons Attribution 4.0 International License]; (B) nanowire strain gauge demonstration [111] [Copyright © 2017 American Chemical Society. This publication is licensed under CC-BY.].

The final type of sensor that has received attention in recent years is the strain gauge nanowire sensor. These sensors exhibit a change in the nanolaser emitted wavelength as a function of the tension or compression the laser is bearing. The first demonstrated strain gauge sensor reported, uses a photonic crystal [109], which, when optically pumped, produces a macroscopic output. The authors found a linear relationship between the strain applied and the nanolaser emission wavelength; compression and tension cause a high-resolution blue and redshift respectively (Figure 32A). This type of nanolaser array has attracted large interest due to their application in a large range of nanoscopic strain analysis [110] (Figure 32).

Another type of strain gauge nanolaser has also been demonstrated by using a single nanowire under reversible tension to induce a wavelength shift [111], this is schematically shown in Figure 32B. The nanowire under tension exhibits a redshift on the emission wavelength. More surprisingly the lasing threshold was not affected when under tension. This provides for the possibility for large scale integration of these strain gauges. Further work has been undertaken on understanding the relationship between cavity modes and induced strain in nanobelts [112].

### 5.2 Biological applications

One field where nanolasers have a wide range of applications is in applied medicine and biological science. Here, nanolasers can be used as biological imaging devices, probing devices, and cell labelling and tracking devices.

#### 5.2.1 Biological probing

A biological probe is a tool that is used to manipulate or study biological entity, this could be a cell, a protein, DNA etc. The most used biological probes are core–shell quantum dots, whose photostability, tunability and bright fluorescence along with their easiness of engineering functional groups to bound to various molecules, have revolutionized fluorescence microscopy [113].

**Figure 33:**
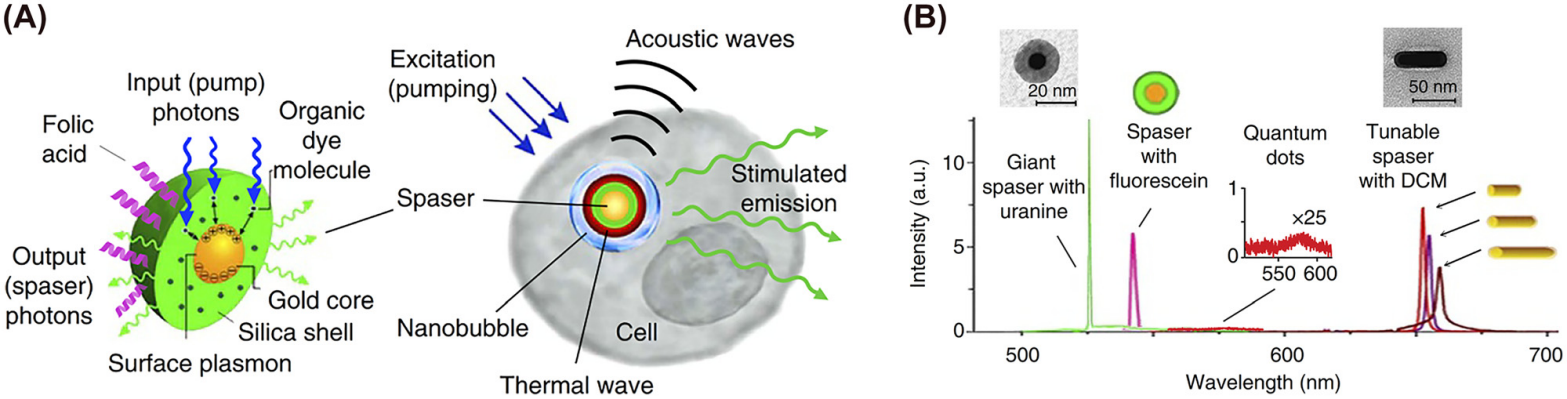
SPASER as a biological probe examples. (A) SPASER schematic [114]; (B) comparison of QD and SPASER emission [114] [Springer Nature, Creative Commons Attribution 4.0 International License].

The SPASER as a biological probe was demonstrated by Galanzha et al. [114] in which an air bubble was introduced around a SPASER with the ability to be injected into living tissue (*ex vivo*) or directly inside of animal cells (*in vitro*), this is schematically shown in Figure 33A. The team found that optical pumping caused stimulated emission at optical powers that did not damage surrounding cells or tissue and produced light that was over 100 times brighter and 30 times narrower than the emission from quantum dots, Figure 33B presents a comparison of the quantum dot and SPASER emission *in vivo*. They further found that functional groups such as folic acid could be attached to the outside of the SPASER, as shown in Figure 33B, to bond with cancer cells. This could be used to target and destroy cancer cells with radiation without damaging the surrounding tissue (Figure 33).

Thermal and acoustic waves were also measured in this landmark experiment, emitted from the SPASER, with possible applications in high resolution in cell imaging and targeted thermal action.

Combining this biomedical technique with wavelength tunability, such as the biocompatible nanorod SPASERs that have recently demonstrated [115], could lead to a new biolabeling and tracking method in cells and should be investigated. Specific wavelength tuning would act as the labelling method while the natural movement of cells would allow to track the particles. Thanks to their high brightness, monochromaticity, and ability to penetrate cells, SPASER could pave the way to smaller, more effective devices than the current microlasers that have been recently demonstrated [116]. The major benefit of this method is that the laser would sit within the cell unlike the current microlaser label-trackers.

#### 5.2.2 STED microscopy (super resolution imaging)

Stimulated emission depletion microscopy (STED), first proposed by Hell and Wichmann [117] is an imaging technique that leads to sub diffraction limited super resolution images. The basic concept involves pumping electrons in a fluorescent nanoparticle into a high energy metastable state, where they would normally undergo spontaneous emission after some time and return to the ground state. However, by sending a second pulse of light with slightly less energy of the transition, before the particles spontaneously decay to the ground state, stimulated emission can be induced, forcing the electron to decay emitting a photon with the same energy of the incoming one. In doing so the area which undergoes stimulated emission will not fluoresce anymore and the effective fluorescence point spread function can be made much smaller than the diffraction limit by using a donut shape depletion beam, in turn generating higher resolution images.

**Figure 34:**
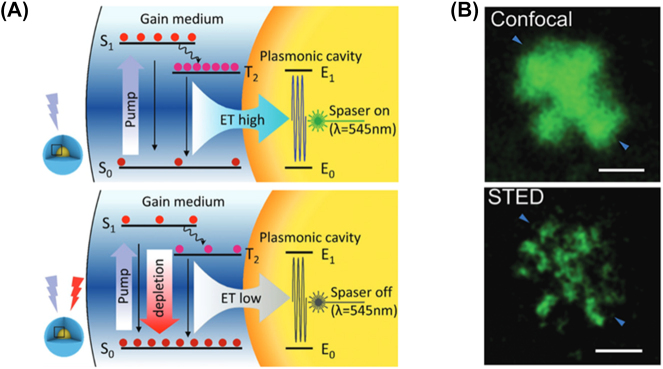
STED microscopy with SPASER. (A) (Top) Common SPASER population inversion and spacing (Bottom) depletion of the SPASER action after the application of the second pulse; (B) (Top) confocal image of SPASERs lasing, (Bottom) increased resolution achieved after the second pulse [118] [© 1999–2023 John Wiley & Sons, Inc.].

This idea has also been implemented by using SPASERs and is known as SPASER-based stimulated emission depletion microscopy [118]. This process works in the same way; electrons are pumped into the upper lasing metastable level by the first pulse in the gain medium of the SPASER, then a second lower energy pulse is then sent to the SPASER before their spontaneous emission lifetime causes them to decay, causing stimulated emission, this is schematically shown in Figure 34A after the first (top) and second (bottom) pulse (Figure 34).

This increase in image resolution can be seen by comparing the confocal and STED images emitted light from SPASER in Figure 34B. The experimenters found that they could increase the optical resolution from 286 nm to 74 nm. This increase in resolution can be applied to a range of sub diffraction limited structures and to generate super resolution images of biological samples.

### 5.3 Photonic and optoelectronic interconnects

One of the most intriguing applications for nanolasers is their integration with or substitution with electronic components in integrated chips. This section will briefly discuss the guiding of laser emission, data storage and transmission, hybrid-optoelectronic circuits, and purely photonic circuits.

#### 5.3.1 Data storage and transmission

To compete with state-of-the-art electronic components nanolasers need to be small, power efficient, and able to transmit large amounts of data among other characteristics. Miller [119] found that to surpass the 2022 projections for electronic integrated circuits set out by the International Technological Roadmap for Semiconductors (ITRS), nanolasers would need to beat an energy consumption rate of 10 fJ/bit at modulation speeds faster than 67.5 GHz. This is promising for nanolasers as demonstrated modulation of nanolasers lays in the terahertz (THz) regime [120] and with ultralow thresholds [121]. However, nanolasers that possess both of these functionalities have not yet been demonstrated. Additionally, more research needs to be undertake on the reliability of nanolaser pulse response characteristics in terms of noise and relaxation time [122].

#### 5.3.2 Directional nanolaser radiation

**Figure 35:**
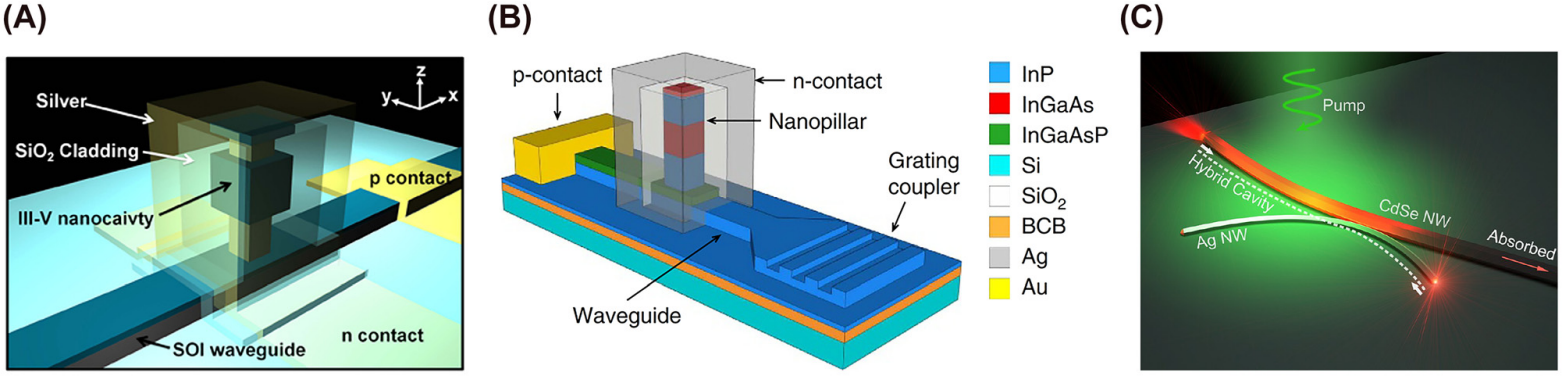
Nanolaser-waveguide coupling examples. (A) Nanocavity coupled to a waveguide [63]. Reproduced with permission [Optica Publishing Group]; (B) nanopillar coupled to a waveguide [125]. [Springer Nature, Creative Commons Attribution 4.0 International License]; (C) photon–plasmon coupled waveguide [124]. Reproduced with permission [ACS Publications].

In reference to the previous section about the viability of nanolasers for data storage and transmission, directionality is a key feature. Nanolaser light in free space suffers from large levels of diffraction as they act as a point like source. One direct advantage is that semiconductor nanolasers have a much higher refractive index than air meaning they act as natural waveguide structures, there has even been discussion over the viability of nanolasers to act as a direct, more compact replacement to optical fibers [123]; moreover they could be easily and directly coupled to integrated photonic waveguides. Many nanolaser waveguide structures have been demonstrated, that attempt to solve the directionality problem, with nanowires [124], nanopillars [125], and nanocavities [125] as just a few examples. Figure 35A presents an electrically pumped nanopillar structure in which light generated in the cavity is coupled and guided through the waveguide (Figure 35).

Figure 35B works in a very similar way to nanocavity, in such that the nanopillar gain medium couples light to the inferior waveguide. And finally, Figure 35C presents duel photonic plasmonic nanowire structure made up of a CdSe semiconductor nanowire separated in space to an Ag nanowire core. When the CdSe nanowire is pumped, plasmonic SPP begins to propagate in the Ag nanowire which emits when they reach the nanowire tip.

#### 5.3.3 Optoelectronic circuitry

If nanolasers can be sufficiently reduced in size, easily coupled with other optical and electrical structures, and maintain stable lasing over long periods of time, they might have an opportunity to be integrated with modern electronic/photonic chip, however a variety of issues stand in the way of this future aim. Optically pumped nanolaser systems are not practical, since they require additional and most of the time macroscopic laser sources for pumping. Hence, electrically driven nanolasers are essential. A variety of nanolasers described in this review have been electrically pumped [54], [55], [58], [60], [61], [62], [126]. However, CMOS compatibility remains one of the major challenges for nanolaser optoelectronic integration. Compatibility with CMOS technology will be crucial for future nanolasers’ integration into optoelectronic circuits; only few examples have been reported to date [80], [127]. Integration must become a major goal in nanolaser research.

#### 5.3.4 Photonic circuits

The idea of full-photonic based integrated circuits was first conceived by Steward E Millar in 1969 and it is only now with the introduction of nanolasers that this idea may be crystallized. Plasmonic nanolasers represent a step towards ultra-confined devices since they act as fully optical light sources with lower pump threshold and power consumption, exhibiting fast modulation devices. Nanolasers have already been shown capable of performing all the logical operations that modern transistors perform [128] and do not suffer from the same communication losses that transistors do [129] making them the ideal device for the next generation of data processing units. Furthermore, removing the need to perform photodetection by converting optical signals to electrical for data communications will further improve the overall efficiency. This ultimate goal, however, will require further research and development toward miniaturization, better control over the propagation of signals, and lower thresholds.

## 6 Perspective

In this monolithic review on the current state of nanolaser research, a wide range of structures, arrays, and applications were examined to determine if nanolasers present a technological revolution or a fundamental dead end. We have shown that nanolasers are very versatile in their design and material configurations, they can be thresholdless, ultrasmall (e.g. SPASERs), directional if in nanowires, readily to be directly coupled with photonics chips, be electrically pumped and operating at room temperature.

The reduced thresholds and mode areas that the theoretical studies on the MHDHA and MHDHM configurations demonstrate, present exciting opportunities for smaller devices that require less power and interact more strongly with the laser gain medium especially when comparing them with the conventional MLDA and MLDLM nanolaser configurations. As can be seen from the plasmonic nanowire section, a substantial amount of work has been focused on changing the gain medium and the geometry of nanowires with the goal of reducing the gain threshold; this has been only somewhat successful. Although theoretically promising, we have not seen any experimental demonstration of the MHDHA and MHDHM configurations yet. These configurations are also appealing since the choice of materials could allow for electrical pumping and potentially directly integrated on chip, coupled to photonic waveguides.

Secondly, more investigations should be undertaken to determine if nanolasers are currently more efficient than bulk lasers. For instance, if the single nanolaser unit has higher efficiency in terms of gain threshold, a nanolaser array could give a nonlinear response, as seen in the previous section, that could exhibit an overall larger laser response than conventional bulk lasers. In this case nanolaser arrays could be utilized also as devices that generate higher coherent optical power than their bulk counterparts.

The wavelength tunability, nanoscopic size, and biocompatibility of nanorod lasers make them ideal for biological applications. We have shown early studies on their suitability as labelling and tracking devices within cells and in tissues. The label can be constructed through wavelength tuning of individual nanorods which can be inserted into cells or into tissue within air bubbles, similarly to that SPASERs have utilized. Furthermore, by attaching specific functional groups to the outside of these nanorods they can be injected into samples in which they bond with the target structures, and then using a bandpass filter, can quickly be identified in a sample providing a novel way to locate specific biological active sites.

Other uses for nanolasers in biological applications could include nanosurgery. The high energy density output of nanolasers could be directed for use in cutting, ablating, or for cauterization in nanosurgery with extreme precision. The ultrafast modulation speeds of nanolasers would allow for high energy pulses that can be applied for extremely short amounts of time improving the precision of this type of surgery.

SPASERS are another area that deserves special attention. From this review it has been clear that these nanolasers are in the order of tens of nanometers in diameter, are biologically compatible, can have a directional emission and are wavelength tunable. The future could see SPASERs at the forefront of data communication, cancer treatment, coupled together in arrays to form macroscopic responses, and a variety of other sensing probing applications. Continued research into SPASERS will likely be imperative in future nanolaser research.

Great strides have been made in nanolaser research in such a young field, since the advent of plasmonic nanolasers miniaturization has been rapid, modulation speeds are increasing, and laser thresholds are decreasing. If these trends continue, fully integrated photonic circuitry, lab-on-a-chip, quantum information processing, bulk lasers built from nanolaser arrays, and non-linear dynamics applications could be at a close horizon.

We have here outlined the tremendous progress and developments reported in the literature especially after 2009, when the first plasmonic nanolaser was experimentally demonstrated and after 2003 when Mark Stockman and David Bergman first proposed the SPASER. There are still challenges before taking nanolasers into the commercial realm, although we believe that future is not far.
